# Proteomic Identification of Small-Subunit Ribosome Assembly Factors in *Trypanosoma brucei*

**DOI:** 10.1021/acs.jproteome.6c00066

**Published:** 2026-05-18

**Authors:** Gustavo Guadagnini Perez, Priscila Mazzocchi Hiraiwa, Verônica Santana da Silva, Nilson Ivo Tonin Zanchin, Beatriz Gomes Guimarães

**Affiliations:** † Carlos Chagas Institute, Oswaldo Cruz Foundation, Rua Prof. Algacyr Munhoz Mader, 3775, 81350-010 Curitiba, Paraná, Brazil; ‡ Biochemistry Postgraduate Program, Federal University of Paraná, 81350-011 Curitiba, Brazil

**Keywords:** ribosome biogenesis, *Trypanosoma brucei*, ribosome small-subunit, affinity purification, proteomic analysis

## Abstract

Trypanosomatid ribosomes display distinctive features, including extensive ribosomal RNA (rRNA) expansions and additional insertions in ribosomal proteins. Moreover, the region corresponding to the human 28S rRNA is fragmented into six molecules in these organisms with a duplication of the 3′ fragment (ε) in*Leishmania*. Although these differences suggest that ribosome biogenesis in trypanosomatids may involve unique processing events, the molecular mechanisms underlying this process are still poorly characterized. In this study, we investigated the protein composition of pre-small-subunit (pre-SSU) complexes in *Trypanosoma brucei*. We generated cell lines expressing tagged versions of UTP6 and PNO1, two conserved ribosome biogenesis factors that provide complementary access to complexes of early SSU processome intermediates and later pre-40S maturation stages. Affinity purification followed by mass spectrometry identified numerous conserved ribosome biogenesis factors alongside a substantial set of trypanosomatid-specific proteins with no assigned function. Structural analyses revealed that many of these uncharacterized proteins contain predicted RNA-binding motifs or protein–protein interaction domains, and have been previously localized to the nucleolus, supporting potential roles in ribosome synthesis. Our findings expand the repertoire of candidate SSU assembly factors in kinetoplastids and highlight species-specific adaptations in ribosome biogenesis, providing a foundation for future functional studies targeting these unique components.

## Graphical abstract

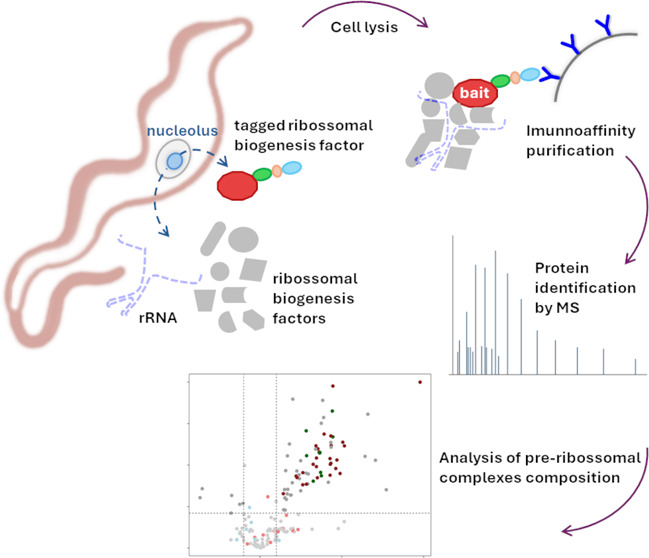

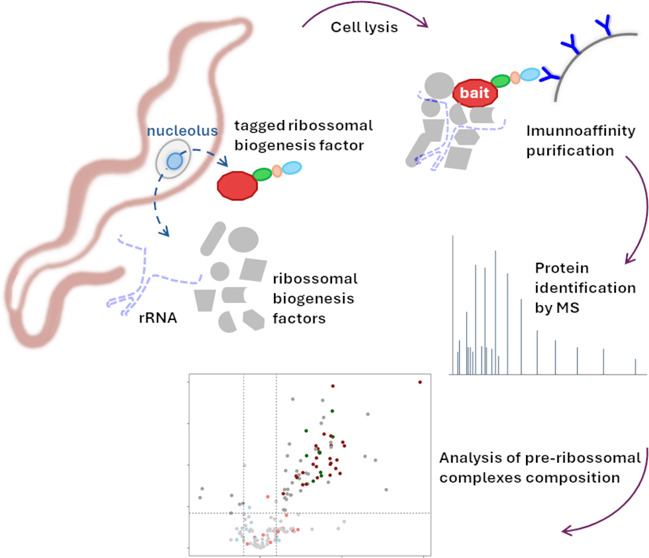

## Introduction

Trypanosomatids are early branching and highly divergent unicellular eukaryotes that include medically important pathogens such as *Trypanosoma brucei*, *Trypanosoma cruzi*, and *Leishmania* spp., the causative agents of severe human diseases. These organisms exhibit numerous molecular peculiarities characteristic of the class Kinetoplastida, which have long posed challenges for researchers. Notably, protein-coding genes in the nuclear genome are arranged in polycistronic transcription units that are transcribed by RNA polymerase II as long pre-mRNA precursors and subsequently processed into mature, monocistronic mRNAs through *trans*-splicing and polyadenylation.^1,2^ In this genome arrangement, individual protein coding sequences are not flanked by RNA polymerase II transcriptional regulatory elements, and they also lack corresponding RNA polymerase II transcription factors. Instead, gene expression in trypanosomatids is regulated predominantly at the post-transcriptional level.^3,4^ During *trans*-splicing, a spliced-leader sequence is added to the 5′ untranslated region of each mRNA; this sequence is initially synthesized as a precursor that undergoes extensive 5′ hypermethylation, resulting in all mature trypanosomatid mRNAs carrying a highly modified 5′ end.^5^

Another distinctive molecular feature of trypanosomatids is the fragmented organization of their rRNA, which reflects their evolutionary origin within the Euglenozoa. For example, in the founding member, *Euglena gracilis*, the large subunit (LSU) rRNA is split into 13 segments in addition to the 5.8S rRNA.^6,7^ Similarly, cytoplasmic rRNA fragmentation occurs in pathogenic trypanosomatids, including *T. brucei*, *T. cruzi*, and *Leishmania* spp. In these organisms, the rRNA homologous to the 25*S*/28S LSU rRNA of other eukaryotes is divided into six fragments, except in *Leishmania* spp., which contains a duplicated segment, resulting in seven fragments.^8–12^

Ribosomes are highly conserved ribonucleoprotein complexes responsible for protein synthesis in all biological systems. Cytoplasmic eukaryotic ribosomes comprise at least four rRNA molecules and approximately 80 ribosomal proteins. In mature ribosomal particles, the rRNAs adopt precisely folded architectures that create binding surfaces for ribosomal proteins, provide platforms for interactions with translation factors, and form the catalytic center that drives peptide synthesis. Assembly of these complex macromolecular structures is energetically demanding and requires tight regulatory coordination during biogenesis.^13,14^

In most eukaryotes, three rRNA species are transcribed by RNA polymerase I as a single precursor molecule that contains the sequences of the mature rRNAs, which are separated by two internal transcribed spacers (ITS1 and ITS2) and flanked by the 5′ and 3′ external transcribed spacers (5′ ETS and 3′ ETS). Processing of this precursor into mature rRNA molecules requires a series of tightly regulated endo- and exonucleolytic cleavages, as well as multiple covalent modifications that occur cotranscriptionally and post-transcriptionally. A fourth rRNA, the 5S rRNA, is transcribed independently by RNA polymerase III from a separate genomic locus and is subsequently incorporated into pre-60S particles as part of the conserved 5S ribonucleoprotein (RNP).^15,16^ Ribosome biogenesis and pre-rRNA processing have been most extensively characterized in *Saccharomyces cerevisiae*. During assembly, rRNA folding and spacer removal are mediated by approximately 200 ribosome biogenesis factors (RBFs)^17,18^ and a similar number of small nucleolar RNAs (snoRNAs), which are short noncoding RNAs that guide site-specific covalent modifications of pre-rRNA, and in the case of U3 snoRNA, also direct early cleavage events.^15,19^

Cryo-electron microscopy studies of the small-subunit (SSU) processome,^20–25^ pre-SSU particles,^26,27^ and pre-large-subunit (pre-LSU) intermediates^28–34^ have elucidated the specific roles of many ribosome biogenesis factors and revealed how pre-rRNA folding, spacer removal, and RBFs association are coordinated during ribosome assembly. These studies provide a structural and mechanistic framework for understanding species-specific variations in ribosome biogenesis.

As mentioned above, the pre-rRNA of trypanosomatids diverges substantially from that of most eukaryotes, containing seven to eight ITSs. LSU pre-rRNA maturation gives rise to six distinct rRNA molecules, in contrast to the single 25*S*/28S rRNA found in other eukaryotes, and is therefore predicted to involve additional processing steps and assembly factors to ensure accurate rRNA maturation.^11,12,35,36^ Moreover, trypanosomatid ribosomes harbor large rRNA expansion segments, which likely impose further constraints on rRNA folding and stabilization.^37,38^ Genome-wide analyses have shown that this increased structural complexity is accompanied by an expanded repertoire of snoRNAs that target a dense pattern of rRNA modifications, consistent with a more complex ribosome biogenesis pathway in trypanosomatids.^35,39,40^ In addition to the removal of external and internal spacers and proper rRNA folding, covalent modifications of pre-rRNA are essential for stabilizing rRNA structure and enabling accurate ribosome assembly. The most abundant and best-characterized modifications are 2′-*O*-methylation (Nm) and pseudouridylation (Ψ), which are directed at specific sites by box C/D and box H/ACA snoRNAs, respectively. Studies in trypanosomatids have demonstrated that specific snoRNAs are required not only for guiding modifications but also for early pre-rRNA cleavage steps, as RNA interference-mediated depletion of individual snoRNAs in *T. brucei* leads to strong accumulation of unprocessed rRNA precursors.^35,41^ Developmentally regulated changes in Ψ modification patterns have been reported in both *T. brucei* and *Leishmania*, indicating functional specialization during life-cycle transitions. Notably, suppression of a single conserved pseudouridine alters translation of specific mRNA subsets and leads to distinct growth defects.^42–44^

Together, these distinctive features indicate that trypanosomatids depend on additional, likely species-specific factors to excise extra internal spacers, stabilize fragmented rRNAs, and correctly fold pre-rRNA transcripts. Such mechanisms may also provide opportunities for the development of selective inhibitors against pathogenic species. However, the molecular pathways underlying ribosome biogenesis in trypanosomatids remain poorly defined, and the identity and function of many candidate RBFs are still unknown. Progress has been limited by the scarcity of studies that isolate and characterize native preribosomal particles, which constrains our understanding of the organization and maturation of pre-SSU and pre-LSU ribosomal intermediates in these organisms.

In this study, we focused on the composition of pre-SSU ribosomal particles in *T. brucei*, aiming to characterize early steps of 18S rRNA maturation. To isolate endogenous particles, we selected two conserved SSU assembly factors, UTP6 and PNO1, as affinity baits. UTP6 was initially identified in yeast as one of the U three proteins (UTPs) associated with Mpp10 and the U3 small nucleolar ribonucleoprotein (snoRNP) and was later assigned to the UTP-B subcomplex of the SSU processome.^45,46^ Structural studies subsequently positioned UTP6 within early SSU assembly intermediates, where it contributes to 5′ ETS recognition and provides scaffolding functions that organize other UTP-B components and facilitate recruitment of the nuclear exosome.^20–25,47,48^ To date, UTP6 has not been characterized in trypanosomatids, and it remains unclear to what extent its functions in SSU assembly are conserved or have diverged in these parasites.

PNO1 was first characterized in yeast as Dim2p, a K homology (KH) domain-containing protein that associates with the endonuclease NOB1 and is required for early steps of SSU biogenesis, including accurate pre-18S rRNA processing.^49^ It was subsequently linked to preribosomal intermediates through its copurification with ENP1 in the SSU processome and pre-40S particles.^50^ Consistent with these roles, PNO1 has been visualized in multiple cryo-electron microscopy structures of the SSU processome and of nucleolar, nucleoplasmic, and cytoplasmic pre-40S intermediates, where it occupies a position adjacent to the decoding site and forms a stable complex with NOB1.^20,21,23–25,47,48,51,52^ Mechanistically, PNO1 restrains NOB1 to prevent premature endonucleolytic cleavage of the 20S pre-rRNA, while also interacting with early rRNA elements that participate in assembly checkpoints, and with elements of the DHR1 helicase and UTP14, preventing premature activation of DHR1.^23^ In *T. brucei*, PNO1 copurifies with the RNA-binding protein ZC3H11, an mRNA stability regulator,^53^ and directly interacts with NOB1, an interaction required for NOB1-dependent pre-rRNA cleavage.^54^ In *Leishmania major*, PNO1 copurifies with NOP56 along with numerous SSU and LSU assembly factors, supporting its conserved involvement in ribosome biogenesis across kinetoplastids.^55^

Using *T. brucei* cell lines expressing tagged versions of UTP6 and PNO1, we performed immunoaffinity purification followed by mass-spectrometry-based proteomic analysis to characterize native pre-SSU particles. These two bait proteins provide complementary temporal windows into SSU biogenesis: TbUTP6 preferentially enriches early nucleolar SSU processome intermediates, whereas TbPNO1 captures both early and later pre-40S particles that progress from the nucleolus to the cytoplasm. This strategy allowed the identification of numerous conserved ribosome biogenesis factors shared with yeast and humans, validating the approach. It also revealed a substantial set of highly enriched trypanosomatid-specific proteins with unknown functions. Together, these findings expand the repertoire of candidate SSU assembly factors in kinetoplastids and establish a foundation for dissecting species-specific mechanisms underlying ribosome biogenesis in these parasites.

## Experimental Section

### Genetic Strategy for Tagging *T. brucei* UTP6 and PNO1

To insert tags into the *T. brucei* endogenous UTP6 and PNO1, we used a strategy based on the pPOT system,^56^ which enables polymerase chain reaction (PCR)-based insertion of tagging cassettes at endogenous loci through long-flanking-homology primers. The pPOTv7 ^57^ and pN–PTP-PURO^58^ vectors were modified to obtain the p2Tag_A (N-terminal Myc-ProtA module) and p2Tag_D (C-terminal SBP-FLAG module) vectors and to enable amplification of two tagging constructs: an SBP-3C-3 × FLAG module (SBP–FLAG), consisting of a streptavidin-binding peptide (SBP), a 3C protease cleavage site, and a triple FLAG epitope; and a 2 × Z Protein A-3 × Myc-TEV-module (Myc–ProtA), comprising two Z-domains derived from *Staphylococcus aureus* Protein A, three Myc epitopes and a Tobacco Etch Virus (TEV) protease site. Primers were designed containing 20 nucleotides matching the tagging cassette and 80 nucleotides flanking the insertion site (Table S1).

The *T. brucei brucei* Lister 427 cell line was used in these experiments.^59^ A first transfection introduced the SBP–FLAG cassette at the 3′ region of the endogenous UTP6 locus. This insertion generates a fusion of the SBP–FLAG module with the C-terminal of the UTP6 protein and this strain was named TbUTP6–SBP–FLAG. After antibiotic selection, accurate insertion of the DNA fragment encoding the SBP-FLAG tag was confirmed by PCR analyses and expression of the TbUTP6-SBP-FLAG protein was confirmed by Western blot using antibodies recognizing the FLAG epitope. The TbUTP6–SBP–FLAG strain served as host for a second transfection to introduce the Myc–ProtA cassette at the 5′ region of the PNO1 locus, which generates a fusion of the ProtA-Myc module to the N-terminal of the PNO1 protein. The resulting strain, expressing both tagged proteins was named TbUTP6–SBP–FLAG + ProtA–Myc–TbPNO1. Similarly, accurate insertion of the DNA fragment encoding the Myc–ProtA tag was confirmed by PCR analyses and expression of the ProtA–Myc–TbPNO1 protein was confirmed by Western blot using antibodies against the Protein A tag. Transfections were performed using 10 μg of PCR products generated by amplification of the tagging cassettes with primers complementary to specific sites in the 3′ and 5′ regions of TbUTP6 and TbPNO1, respectively. A total of 5 × 10^7^ logarithmic-phase growing cells resuspended in BSF buffer (5 mM KCl, 0.15 mM CaCl_2_, 90 mM Na_2_HPO_4_, 50 mM HEPES pH 7.3) were used, followed by electroporation on a Lonza Nucleofector 2b using the X-001 program. Cells were recovered in selective medium containing 2 μg/mL puromycin or blasticidin, depending on the genetic marker of the tagging cassette used in the transfection.

Control cell lines expressing the Green Fluorescent Protein (GFP) fused to Myc–ProtA or SBP–FLAG were generated independently, by integrating pGFP2Tag_A and pGFP2Tag_B, respectively, in the *T. brucei* genome at a nontranscribed spacer of the rDNA repeat. These vectors are derived from the pTbFIX plasmid.^60^ Cells expressing either GFP–Myc–ProtA or GFP–SBP–FLAG were processed in parallel as controls for affinity purification and mass spectrometry analyses.

### Analysis of Tag Insertion by PCR and by Western Blot

PCR was prepared using 100 ng of *T. brucei* genomic DNA and 0.5 μM of each primer (Table S2) and performed on a ProFlex PCR system (Thermo Fisher Scientific, Marsiling, Singapore). The reaction products were visualized using a 1% agarose gel. For immunodetection, cell extracts corresponding to 5 × 10^6^ cells expressing TbUTP6–SBP–FLAG or TbUTP6–SBP–FLAG + ProtA–Myc–TbPNO1, or 1 × 10^6^ cells expressing GFP–Myc–ProtA or GFP–SBP–FLAG, were fractionated by SDS-PAGE and transferred to polyvinylidene fluoride (PVDF) membranes. The membranes were incubated with mouse anti-FLAG M2 (Sigma-Aldrich F1804; 1:2400 dilution) or rabbit anti-Protein A (Sigma-Aldrich P3775; 1:40,000 dilution), followed by Alexa Fluor 680-conjugated goat antimouse IgG (Invitrogen A-21058; 1:10,000 dilution) or IRDye 800CW-conjugated donkey antirabbit IgG (LI-COR 926-32213; 1:15,000 dilution). Fluorescence images were acquired on a LI-COR Odyssey imaging system.

### Cell Culture and Immunoaffinity Purification

Procyclic trypomastigotes of the *T. brucei brucei* Lister 427 derived strains were cultivated at 28 °C in SDM-79 medium supplemented with 10% heat-inactivated fetal bovine serum and the appropriate selection antibiotics (puromycin, 5 μg/mL; blasticidin, 10 μg/mL). Three independent biological replicates were grown and harvested at 1 × 10^10^ cells per replicate during logarithmic phase. GFP-tagged control lines carrying the same epitope tag as the bait-expressing strains were processed in parallel using identical procedures.

Cells were pelleted by centrifugation at 5000*g* for 10 min at 4 °C, washed three times with phosphate-buffered saline, and stored at −80 °C until use. Frozen cells were thawed on ice and resuspended at a 1:4 ratio in lysis buffer (20 mM Tris–HCl pH 7.4, 100 mM NaCl, 1 mM MgCl_2_, 0.05% Triton X-100, 5% glycerol, 1 mM dithiothreitol (DTT), and 1× cOmplete Protease Inhibitor Cocktail, (Roche #04693116001). Cells were lysed using a Dounce homogenizer, and lysates were clarified twice by centrifugation: first at 2000*g* for 15 min, then at 12,000*g* for 20 min, both at 4 °C.

Clarified extracts were incubated for 2 h at 4 °C with 20 μL of packed resin, using either IgG Sepharose 6 Fast Flow (Cytiva 17-0969-01) for extracts from GFP–Myc–ProtA and TbUTP6–SBP–FLAG + ProtA–Myc–TbPNO1 cells, or Anti-FLAG M2 affinity resin (Merck M8823) for extracts from GFP–SBP–FLAG and TbUTP6–SBP–FLAG cells. All resins were pre-equilibrated with buffer A (20 mM Tris–HCl pH 7.4, 100 mM NaCl, 1 mM MgCl_2_, 0.05% Triton X-100, 5% glycerol). After five washes with buffer A, elution was performed using 4.75 ng/μL TEV protease for 1 h at 22 °C (Myc–ProtA-tagged samples) or 150 ng/μL 3 × FLAG peptide (Sigma F4799) for 2 h at 4 °C (SBP–FLAG-tagged samples).

### Mass Spectrometry Sample Preparation and Data Acquisition

Immunoprecipitated samples were processed for in-gel digestion as described previously.^61^ Briefly, samples concentrated in a SpeedVac were resolved on a 10% SDS-PAGE gel, and each lane was excised and finely chopped. Gel pieces were subjected to sequential dehydration and rehydration steps, including reduction with DTT, alkylation with iodoacetamide, digestion with trypsin, and peptide extraction with acetonitrile. Peptides were desalted using C18 StageTips and stored at 4 °C until Liquid Chromatography–Tandem Mass Spectrometry (LC–MS/MS) analysis.

Prior to injection, peptides were eluted from StageTips with 0.1% formic acid in 40% acetonitrile. Samples derived from GFP–Myc–ProtA and ProtA–Myc–TbPNO1 strains were analyzed on a Vanquish Neo Ultra-High Performance Liquid Chromatography system coupled to an Orbitrap Exploris 480 (Thermo Fisher Scientific), whereas samples from GFP–SBP–FLAG and TbUTP6–SBP–FLAG strains were analyzed on an Orbitrap Fusion Lumos system (Thermo Fisher Scientific). Peptides were separated by nano–liquid chromatography using mobile phase A (0.1% formic acid in water) and mobile phase B (0.1% formic acid in 95% acetonitrile) at 250 nL/min. Peptides were eluted over a linear gradient from 5% to 25% B in 55 min, followed by an increase to 40% B over 5 min. The analytical column measured 15 cm × 75 μm (length x diameter) and was packed with 3 μm C18 resin.

Mass spectra were acquired in positive ion mode with a spray voltage of 2300 V and an ion transfer tube temperature of 275 °C. Full MS scans were collected in the Orbitrap at a resolution of 120,000 over an *m*/*z* range of 350–1200 (Myc–ProtA samples) or 300–1500 (SBP–FLAG samples). The radio frequency (RF) lens was set to 50% (Myc–ProtA samples) or 30% (SBP–FLAG samples). The automatic gain control (AGC) target was 300% for Myc–ProtA samples or 100% for SBP–FLAG samples, with maximum injection times of 25 ms (Myc–ProtA samples) or 50 ms (SBP–FLAG samples). Internal calibration was enabled using EASY-IC (Thermo Fisher Scientific).

For GFP–Myc–ProtA and ProtA–Myc–TbPNO1, data-dependent acquisition (DDA) was configured to fragment the top 20 most intense precursor ions. For GFP–SBP–FLAG and TbUTP6–SBP–FLAG, a 2 s cycle time was used between master scans. Precursors with charge states of 2–6 (Myc–ProtA) or 2–7 (SBP–FLAG) were selected using intensity thresholds of 8 × 10^3^ or 2 × 10^4^, respectively. Dynamic exclusion was set to 30 s (Myc–ProtA) or 60 s (SBP–FLAG) with a tolerance of 10 ppm. Precursor ions were isolated with a window of 2.0 *m*/*z* (Myc–ProtA) or 1.6 *m*/*z* (SBP–FLAG) and fragmented by higher-energy collisional dissociation (HCD) with normalized collision energy of 30%. MS/MS spectra were acquired in the Orbitrap at a resolution of 15,000, with an AGC target of 50% and an automated maximum injection time (Myc–ProtA) or an AGC target of 100% and maximum injection time of 22 ms (SBP–FLAG).

### Proteomic Data Processing and Bioinformatic Analysis

Since the *T. brucei brucei* Lister 427 was used in the experiments, the LC–MS/MS data were processed with MaxQuant v2.7 ^62^ using the *T. brucei brucei* Lister 427 reference proteome (2018 release) as the search database.^63^ Subsequently, the list of identified proteins was used to retrieve the proteins Ids from the *T. brucei brucei* TREU927 reference genome. Label-free quantification (LFQ) was enabled, and Match Between Runs was applied with default settings. The resulting proteinGroups file was further processed in R. Protein groups were filtered according to stringent criteria to minimize false-positive identifications. Proteins were retained only if they met all of the following thresholds: (i) mean LFQ intensity ratio between bait IP and GFP control ≥2 (mean LFQi_bait_/mean LFQi_GFP_ ≥2); (ii) mean MS/MS count ratio ≥2; (iii) sequence coverage of the bait protein ≥10% in each replicate; (iv) MaxQuant score ≥10; and (v) LFQi > 0 in all bait replicates.

LFQ values equal to zero in GFP controls were imputed using the lowest nonzero LFQ value across all replicates. For statistical analysis, LFQ intensities were log_2_-transformed, and pairwise comparisons between bait and GFP control samples were performed using a two-sample *t*-test with Benjamini–Hochberg correction and false discovery rate (FDR) ≤ 0.05. Proteins with adjusted *p* < 0.05 were considered significantly enriched.

Identified proteins were queried by BLASTp against *S. cerevisiae* (S288C, R64), *Homo sapiens* (GRCh38.p14), *T. cruzi* (Dm28c), *L. major* (Friedlin), and *Leishmania braziliensis* (MHOM/BR/75/M2904) proteomes. The best-scoring hit, based on alignment coverage, percentage of positives, and E-value, was used to assign putative orthologs. To assess structural conservation, predicted structures were retrieved from the AlphaFold Database^64^ or generated using the AlphaFold Server^65^ for all proteins annotated as ribosome biogenesis-related, as well as for proteins of unknown function. Each structure was aligned to its closest ortholog using TM-align.^66^ Structural similarity was evaluated based on TM-score, alignment coverage, and percent sequence identity. For proteins lacking orthologs in yeast or humans, AlphaFold-predicted structures^64,65^ were further analyzed for structural homology using FoldSeek^67^ and the CATH-Gene3D.^68^ Three-dimensional structures were visualized using UCSF ChimeraX.^69^

## Results and Discussion

### Immunoprecipitation of Tagged *T. brucei* UTP6 and PNO1 Copurifies Conserved Ribosome Biogenesis Factors

To characterize protein components of *T. brucei* preribosomal intermediates, we generated cell lines expressing tagged UTP6 (TbUTP6–SBP–FLAG) and PNO1 (ProtA–Myc–TbPNO1). Cell lines expressing GFP fusions bearing the same tags (GFP–SBP–FLAG or GFP–Myc–ProtA) served as controls. Integration of the nucleotide sequences encoding the SBP–FLAG and ProtA–Myc tags at the correct positions in the *T. brucei* genome was confirmed by PCR analyses and expression of the tagged versions of UTP6 and PNO1 was confirmed by Western blot (Figure S1). Following selection using the puromycin genetic marker, expression of tagged GFP (GFP–SBP–FLAG and GFP-Myc–ProtA) was also confirmed by Western blot (Figure S1). Each tagged protein and its corresponding GFP control was subjected to affinity purification, using anti-FLAG resin for SBP–FLAG–tagged proteins or IgG-Sepharose for ProtA–Myc–tagged proteins.

LC–MS/MS analysis of in-gel digested samples identified 388 and 2782 proteins in the TbUTP6 and TbPNO1 pull-downs, respectively. These numbers include all low abundance proteins, with very low peptide counts, some even not present in all replicates, most falling below the statistical threshold as seen in Figure S2. After removal of contaminants, stringent filtering, and statistical testing, 49 and 207 proteins were significantly enriched in the TbUTP6 and TbPNO1 pull-downs, respectively (Figure 1 and Tables S3 and S4). Among these, conserved ribosome biogenesis factors accounted for ∼45% of proteins enriched with TbUTP6 and ∼19% of those enriched with TbPNO1.

**Figure 1:**
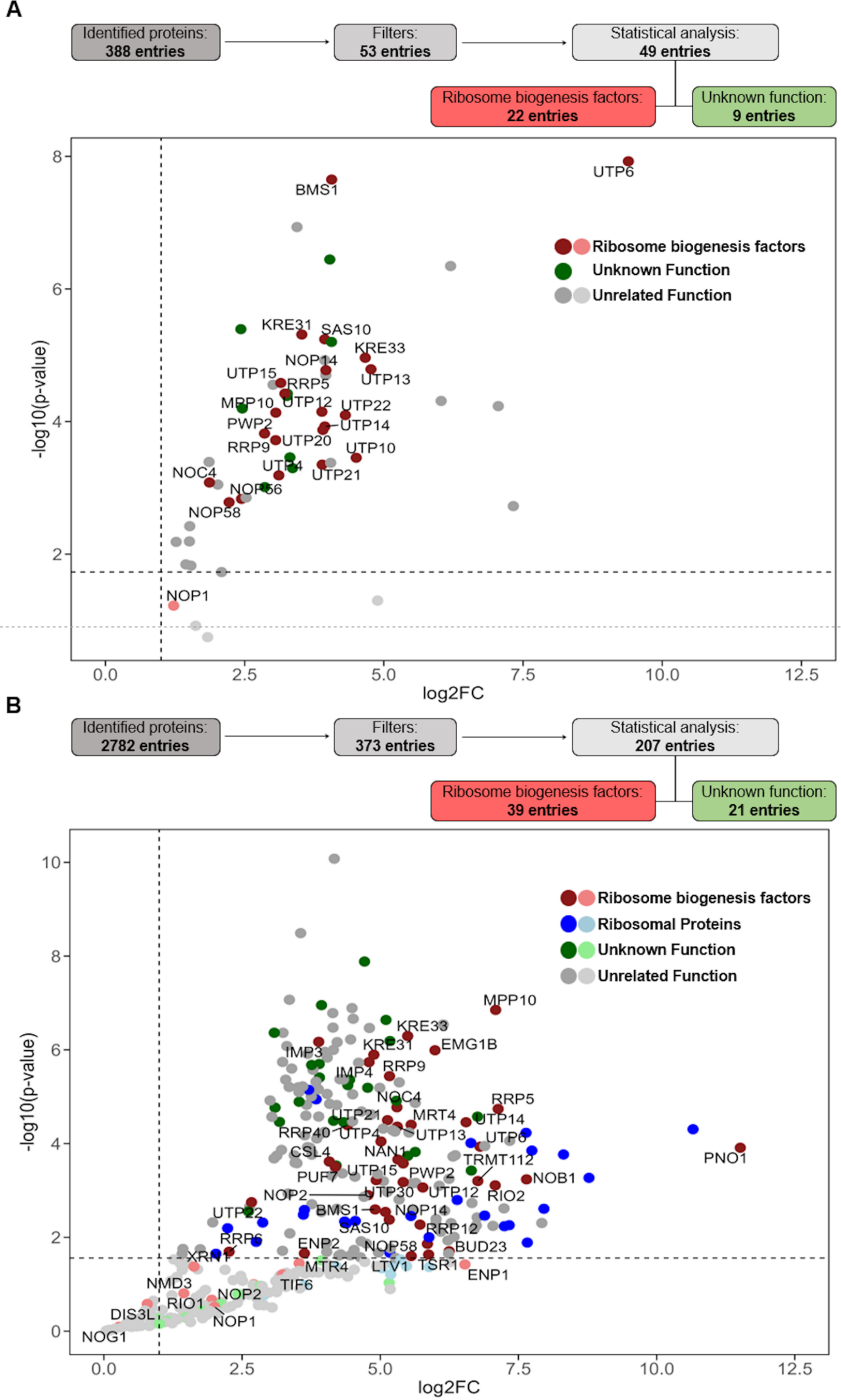
Mass spectrometry analysis of proteins enriched by immunoprecipitation using *T. brucei*UTP6 (A) and PNO1 (B) as baits. For each bait, the upper panel shows an overview of the data-processing workflow, indicating the number of proteins identified, retained after filtering, and significantly enriched in UTP6- (A) or PNO1-based (B) immunoprecipitations. The lower panel shows the corresponding volcano plots, highlighting proteins differentially enriched relative to the GFP-tagged controls. Data are displayed as log_2_ fold change (log_2_ FC) (*x*-axis) versus −log_10_ *p*-value (*y*-axis). Significance thresholds correspond to adjusted *p* ≤ 0.05 (FDR ≤0.05) and log_2_ FC > 1. Conserved ribosome biogenesis factors are displayed in red (significant) or pink (not significant) and annotated by their yeast orthologs; ribosomal proteins appear in blue (significant) or cyan (not significant); proteins of unknown function appear in green (significant) or light green (not significant). Proteins of unrelated function are indicated in gray (significant) and light gray (not significant).

Several conserved factors involved in early SSU biogenesis were identified (Figures 2 and 3). Three were detected exclusively in the TbUTP6 pull-down, whereas 20 were common to both immunoprecipitations. In addition, 18 factors were identified only in the TbPNO1 pull-down, including five proteins previously characterized as acting at later stages of SSU maturation or in LSU biogenesis (see next section for details). Consistent with an early nucleolar role, TbUTP6 yielded a relatively compact and compositionally homogeneous set of interactors, whereas TbPNO1 associated with a more complex and heterogeneous ensemble reflective of its activity across nucleolar, nucleoplasmic, and cytoplasmic stages.

**Figure 2:**
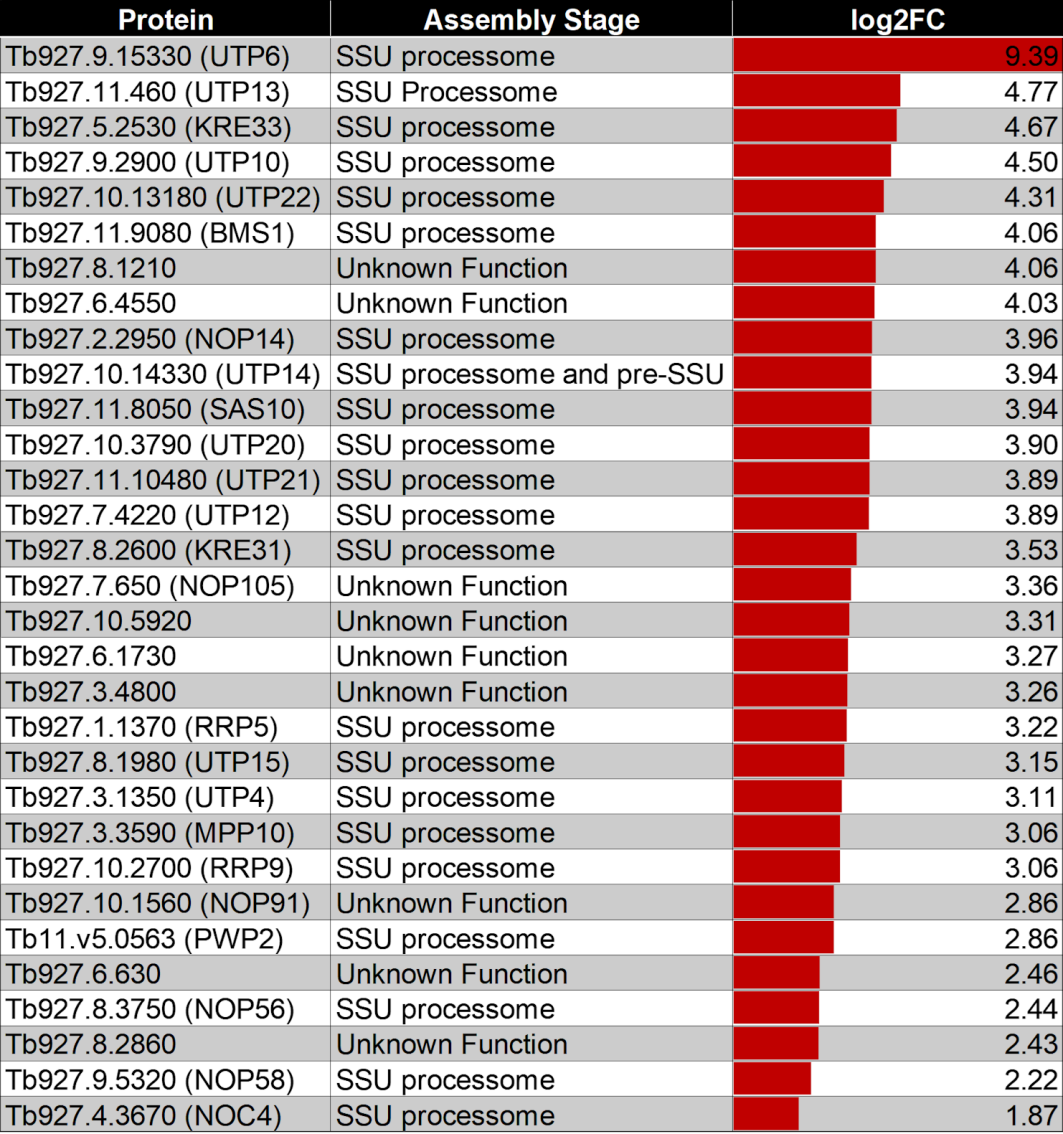
Ribosome biogenesis factors and proteins of unknown function enriched with *T. brucei* UTP6. TriTrypDB accession numbers for *T. brucei* proteins are shown together with the corresponding names of conserved ribosome biogenesis factors (closest homologs in yeast or humans). Proteins of unknown function are indicated by accession numbers only, with the exception of a few *T. brucei* proteins that have been preliminarily characterized but lack a defined role in ribosome biogenesis. Assembly stage assignments were based on structural and biochemical studies of preribosomal complexes in yeast and humans.^20–26,46^ Log_2_ fold-change (log_2_ FC) values were calculated as described in the Experimental Section. Bars represent normalized log_2_ FC, with the value for the bait protein set as maximum, and 0 as minimum.

**Figure 3:**
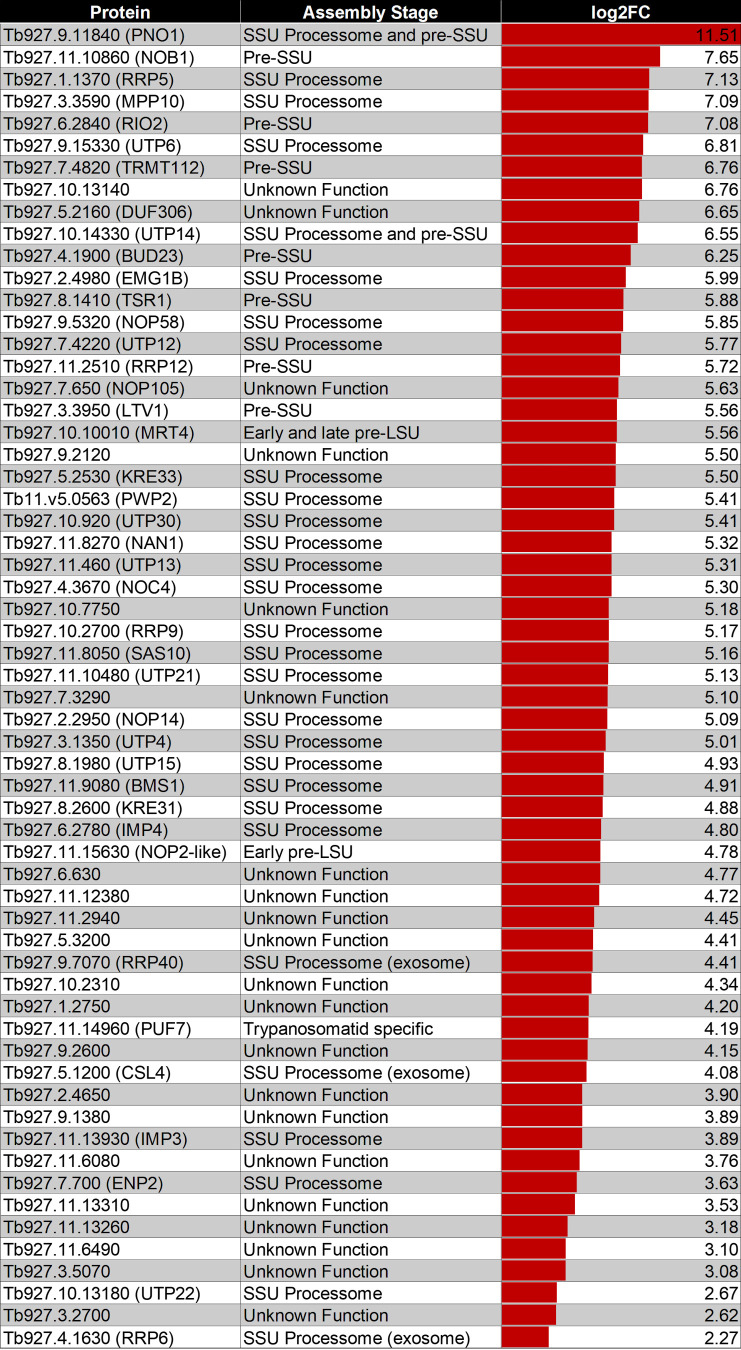
Ribosome biogenesis factors and proteins of unknown function enriched with *T. brucei* PNO1. TriTrypDB accession numbers for *T. brucei* proteins are shown together with the corresponding names of conserved ribosome biogenesis factors (closest homologs in yeast or humans). Proteins of unknown function are indicated by accession numbers only, with the exception of a few *T. brucei* proteins that have been preliminarily characterized but lack a defined role in ribosome biogenesis. Assembly stage assignments were based on structural and biochemical studies of preribosomal complexes in yeast and humans.^20–27,33,34,45–48,51^ Log_2_ fold-change (log_2_ FC) values were calculated as described in the Experimental Section. Bars represent normalized log_2_ FC, with the value for the bait protein set as maximum, and 0 as minimum.

### Comparative Analysis Reveals Conserved Components of Early Preribosomal Intermediates

To situate these findings within established preribosomal intermediates, we compared the identified *T. brucei* orthologs with known components of the SSU processome^20–25,47,48^ and early pre-SSU complexes^26,27^ from *S. cerevisiae* and *H. sapiens*, compiled from structural studies, and categorized these orthologs according to their stage of association with preribosomal particles (Table 1). Based on this comparison, six major groups emerged: (1) proteins enriched exclusively in TbUTP6 pull-down; (2) proteins enriched in both TbUTP6 and TbPNO1 pull-downs; (3) proteins enriched exclusively in TbPNO1 pull-down; (4) proteins enriched with TbPNO1 but primarily involved in later cytoplasmic SSU steps or in LSU biogenesis; (5) proteins detected prior to filtering but not retained after statistical analysis; and (6) proteins lacking identifiable homologs.

**Table 1:** Conservation and Absence of SSU Processome and Early Pre-SSU Components Detected in *T. brucei* UTP6 and PNO1 Pull-Down**s** ^a^

					IP UTP6			IP PNO1			
	SSU processome		early pre-SSU		*T. brucei*			*T. brucei*			
protein	*S. cerevisiae*	*H. sapiens*	*S. cerevisiae*	*H. sapiens*	stat	filter	original data	stat	filter	original data	group
NOP56/NOL5A	X	X			X				X		**1**
UTP20	X	X			X					X	
HEATR1/UTP10	X	X			X					X	
MPP10/MPHOSPH10	X	X	X		X			X			**2**
WDR46/UTP7/KRE31	X	X			X			X			
RRP9/RNU3IP2	X	X			X			X			
EXOSC10/RRP6	X	X			X			X			
PWP2/UTP1	X	X	X		X			X			
WDR36/UTP21	X	X	X		X			X			
UTP15	X	X			X			X			
UTP4/CIRH1A	X	X			X			X			
UTP14A/UTP14	X	X	X	X	X			X			
NAT10/ALP/KRE33	X	X			X			X			
NOL6/UTP22	X	X	X		X			X			
SAS10/UTP3	X	X	X		X			X			
UTP6	X	X			X			X			
WDR3/UTP12/DIP2	X	X	X		X			X			
NOP58	X	X			X			X			
NOP14/UTP2	X	X	X		X			X			
BMS1	X	X	X		X			X			
NOC4L/NOC4/UTP19	X	X	X		X			X			
RRP5	X				X			X			
UTP13/TBL3/SAZD	X		X		X			X			
WDR75/UTP17/NAN1	X	X					X	X			**3**
EMG1/NEP1	X	X	X					X			
IMP4/BXDC4	X	X	X					X			
NOL10/ENP2	X	X					X	X			
PNO1	X	X	X	X				X			
IMP3	X	X	X					X			
UTP30	X						X	X			
BUD23/MERM1				X				X			
TSR1				X				X			
RRP12				X			X	X			
RRP40/EXOSC3	X							X			
CSL4/EXOSC1	X							X			
TRMT112				X				X			
NOB1	Late Pre-SSU	late pre-SSU	late pre-SSU	late pre-SSU				X			**4**
RIO2	late pre-SSU	late pre-SSU	late pre-SSU	late pre-SSU				X			
LTV1	late pre-SSU	late pre-SSU	late pre-SSU	late pre-SSU				X			
MRT4	pre-LSU	pre-LSU	pre-LSU	pre-LSU				X			
NOP2	pre-LSU	pre-LSU	pre-LSU	pre-LSU				X			
NOP1	X	X				X			X		**5**
SOF1/DCAF13/WDSOF1	X	X					X			X	
UTP11	X	X					X			X	
UTP18	X	X					X			X	
MTR4/DOB1	X						X		X		
BFR2/AATF/CHE1/DED	X	X					X		X		
RCL1/RNAC/RPC2	X	X	X				X			X	
ENP1/BYSL	X		X	X			X		X		
RRP43/EXOSC8	X									X	
KRR1/HRN2	X	X								X	
DIM1/DIMT1	X	X	X	X						X	
SKI6/RRP41/EXOSC4	X									X	
ECM16/DHR1/DHX37/DDX37	X	X	X							X	
FCF1/UTP24	X	X	X							X	
RRP45/EXOSC9	X									X	
DIS3/RRP44	X									X	
SNU13	X	X								X	
SLX9/C21orf70				X						X	
RRP7	X	X	X		no *T. brucei* homolog			no *T. brucei* homolog			**6**
RRP4/EXOSC2	X				no *T. brucei* homolog			no *T. brucei* homolog			
LRP1	X				no *T. brucei* homolog			no *T. brucei* homolog			
UTP9	X				no *T. brucei* homolog			no *T. brucei* homolog			
LCP5	X				no *T. brucei* homolog			no *T. brucei* homolog			
RRT14	X				no *T. brucei* homolog			no *T. brucei* homolog			
FAF1	X				no *T. brucei* homolog			no *T. brucei* homolog			
MTR3/EXOSC6	X				no *T. brucei* homolog			no *T. brucei* homolog			
RRP46/EXOSC5	X				no *T. brucei* homolog			no *T. brucei* homolog			
UTP8	X				no *T. brucei* homolog			no *T. brucei* homolog			
MPP6	X				no *T. brucei* homolog			no *T. brucei* homolog			
WDR43/UTP5	X	X			no *T. brucei* homolog			no *T. brucei* homolog			
BUD21/UTP16/NOL7	X	X			no *T. brucei* homolog			no *T. brucei* homolog			
FCF2/TDIF2/DNTTIP2/ERBP	X	X			no *T. brucei* homolog			no *T. brucei* homolog			
RRP42/EXOSC7	X				no *T. brucei* homolog			no *T. brucei* homolog			
AROS/RPS19BP1		X			no *T. brucei* homolog			no *T. brucei* homolog			
FSAF1/C1orf131/cPERP-A		X			no *T. brucei* homolog			no *T. brucei* homolog			
NGDN/C14orf120		X			no *T. brucei* homolog			no *T. brucei* homolog			

a Proteins detected in the TbUTP6 and TbPNO1 pull-downs were compared with established components of the SSU processome^20–25,47,48^ and early pre-40S particles^26,27^ from *S. cerevisiae* and *H. sapiens*. For the *T. brucei* datasets, three detection categories are indicated: statistically enriched (Stat), retained after filters application (Filter), and identified prior to filtering (Original data). Orthologs corresponding to factors acting at later stages of SSU or LSU maturation in *S. cerevisiae* are indicated as Late Pre-SSU and Pre-LSU, respectively. Proteins lacking identifiable homologs in *T. brucei* are also indicated. Proteins are further classified into Groups 1–6, as described in the main text. For proteins in group 4, specific references are NOB1,^24,26^ RIO2,^48^ LTV1,^26,27^ MRT4,^28,33,34^ and NOP2.^33^

These groupings reveal partially conserved trajectories of SSU assembly in *T. brucei*, while also highlighting divergences that may correspond to parasite-specific processing events. For instance, conserved core components of the C/D snoRNP responsible for 2′-*O*-methylation, such as NOP56 and NOP58,^70–72^ were readily detected, along major structural RBFs including NOC4,^21^ RRP5,^73^ UTP10,^74^ and UTP20.^20^ In contrast, several conserved factors typically associated with early preribosomal intermediates, such as MTR4 ^75^ and FCF1,^76^ were detected only in the original data sets. Such discrepancies may reflect transient or low-abundance interactions, narrow temporal windows of engagement, or differences in the architecture and dynamics of trypanosomatid preribosomes relative to their yeast and human counterparts.

Comparison between TbUTP6 and TbPNO1 pull-downs further revealed distinct enrichment patterns. Nuclear exosome components RRP6, EXOSC1, EXOSC3 ^21,77^ were detected exclusively in the TbPNO1 pull-down, together with factors acting at later stages of SSU maturation (NOB1, RIO2, LTV1),^26,27^ proteins involved in LSU biogenesis (MRT4, NOP2),^30,33,78^ and the trypanosome-specific factor PUF7.^79^ By contrast, the few RBFs detected exclusively in the TbUTP6 pull-down comprised two large structural factors (UTP10 and UTP20),^20,74^ as well as the box C/D snoRNP component NOP56.^70–72^

These observations reinforce the idea that, although the overall mechanism of SSU maturation is conserved, the *T. brucei* system includes species-specific adaptations in both factor composition and assembly timing. Furthermore, the contrasting interactomes of TbUTP6 and TbPNO1 reflect their distinct positions along the SSU maturation pathway.

### Proteins Not Commonly Involved in Ribosome Biogenesis

Interestingly, proteins not commonly associated with ribosome biogenesis were also enriched in the TbPNO1 pull-down, suggesting that some may participate in regulatory or structural pathways that intersect with ribosome production. For example, KKIP6, a kinetoplastid-specific kinetochore-associated protein, was enriched more than 4.6-fold (log_2_ fold change relative to GFP-tagged controls). Unlike previous reports describing cross-linking between KKIP6 and KKIP1 and interactions with additional kinetochore components,^80,81^ KKIP6 was independently enriched in our data set. In addition, ZC3H41 (Z41AP), enriched more than 6.8-fold, is an RNA helicase–containing regulator of ribosomal protein mRNAs.^82,83^ Although these proteins are not canonical ribosome biogenesis factors, their robust enrichment combined with their predominantly nuclear localization supports potential roles in RNA metabolism or in coordinating ribosome biogenesis with cell-cycle progression.

Several additional proteins involved in cytoskeletal and cytokinesis regulation, including KIF9,^84^ MARP2,^85^ CAAP1,^86^ p25-alpha (TPPP2),^87^ and CMF15 (CFAP52),^88,89^ were detected in either the TbUTP6 or the TbPNO1 pull-downs, many of which are trypanosomatid-specific. These factors have previously been shown to colocalize with spindle-associated proteins during closed mitosis in trypanosomes,^90^ a context in which the nucleolus remains intact. This spatial proximity suggests a potential coordination between ribosome production and cell-cycle progression, supporting the notion that these processes may be mechanistically linked. In line with this, studies in *S. cerevisiae* have shown that depletion of individual ribosome biogenesis factors can lead to specific defects in cytokinesis and spindle organization and, in some cases, may affect cell-cycle progression prior to detectable changes in ribosome abundance or translational capacity.^91–93^

Protein quality-control and turnover pathways also intersect with ribosome biogenesis.^94^ At least two prefoldin subunits^95^ were detected in the TbPNO1 pull-down, together with the proteasome subunit PSMB5 ^96,97^ and the ubiquitin-conjugating enzyme UBE2E1.^98^ Some ribosomal proteins are ubiquitinated during biogenesis, and these modifications act as chaperone-like signals essential for proper pre-rRNA processing.^99,100^ Thus, the enrichment of these proteins suggests that trypanosomatids may rely on tight control of protein folding and turnover during ribosome assembly.

### Structural Conservation and Diversity among Ribosome Biogenesis Factors

To assess the extent of structural conservation among ribosome biogenesis factors detected in the TbUTP6 and TbPNO1 pull-downs, we retrieved predicted structures from the AlphaFold Database^64^ or generated structural models using the AlphaFold Server,^65^ and compared them to predicted or experimentally determined structures of orthologs from yeast and humans using TM-align^66^ (Figure 4A). Conservation within kinetoplastids was also evaluated by comparing each *T. brucei* RBF to its orthologs in *T. cruzi*, *L. major*, and *L. braziliensis* (Figure 4B). Many conserved RBFs displayed intermediate to high structural similarity across species (TM-score ≥0.5). These included methylation-related proteins such as NOP56, NOP58 ^70–72^ and TRMT112,^101^ the RNA helicase KRE33,^102^ and several proteins characterized by canonical RNA– or protein–interaction architectures, including leucine-rich repeats (NOC4,^21^ UTP13 ^21,45^ ) and β-propeller-containing factors (UTP15,^20,47^ PWP2 ^22,70,103^ ).

**Figure 4:**
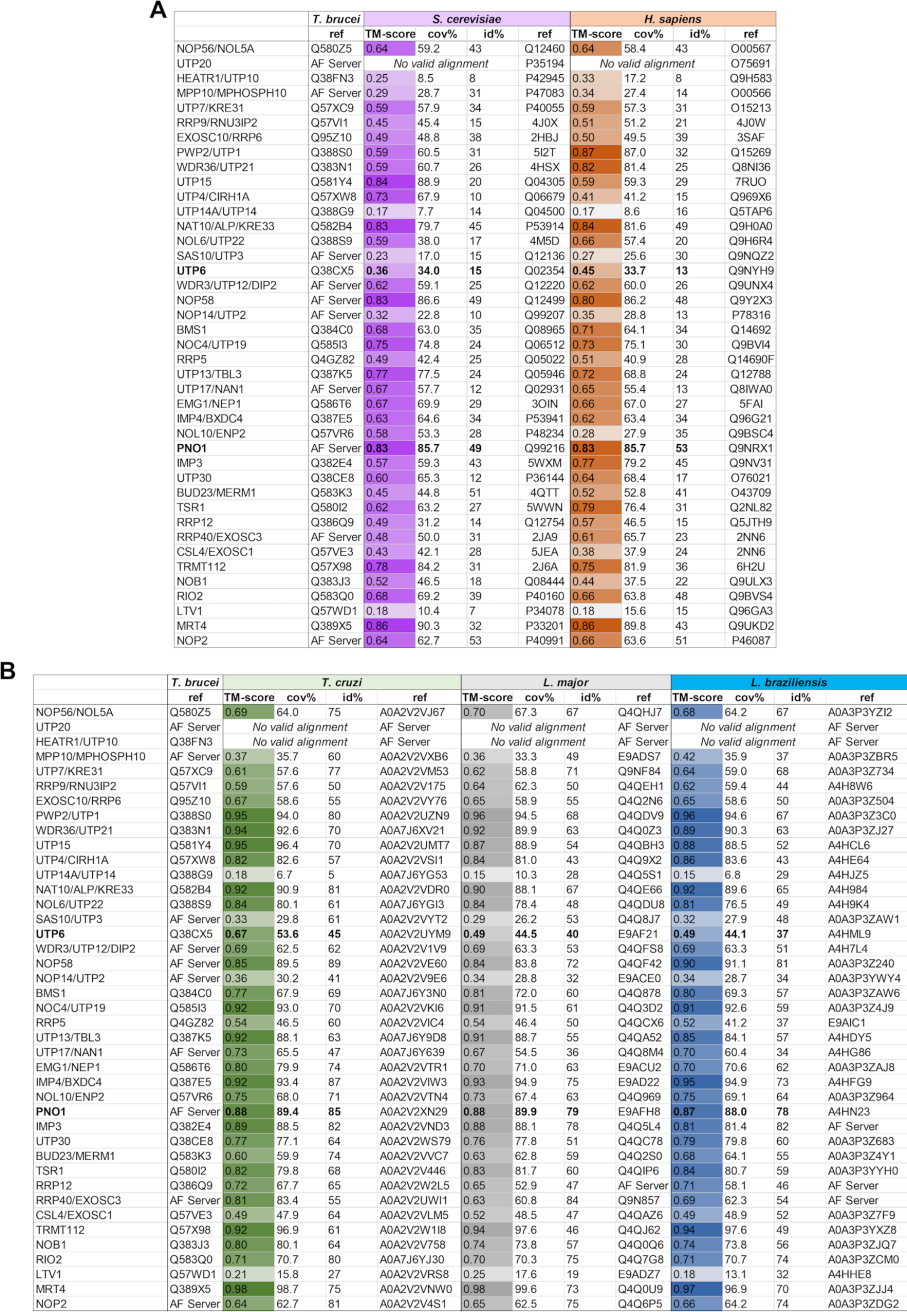
Analysis of structural conservation of enriched ribosome biogenesis factors across kinetoplastids, yeast, and humans. Predicted structures from AlphaFold Database, generated using AlphaFold server (AF Server) or available experimental structures in the Protein Data Bank (PDB) (as indicated by the codes in the column “ref”) were aligned using TM-align to evaluate the structural conservation of *T. brucei* ribosome biogenesis factors enriched in UTP6 or PNO1 pull-downs. Each *T. brucei* protein was compared with its closest ortholog in *S. cerevisiae* (S288C) and *H. sapiens* (GRCh38.p14) (A); and *T. cruzi* (Dm28c), *L. major* (Friedlin) and *L. braziliensis* (MHOM/BR/75/M2904) (B). TM-score, alignment coverage (cov %), and sequence identity (id %) are indicated. Color scales indicate relative TM-score values (light colors = low conservation; dark colors = high conservation). Cases in which TM-align could not compute a valid alignment are indicated. TbUTP6 and TbPNO1 are highlighted in bold.

One of our baits, TbPNO1, showed high structural conservation (TM-scores greater than 0.8) across all species examined, except for its unstructured N-terminal region, as illustrated in Figure S3. This strong conservation is consistent with its essential and structurally constrained roles in early SSU maturation, including the proper folding of 18S rRNA and the regulation of late NOB1-mediated cleavage.^20,27^ Importantly, NOB1 and PNO1 have been previously characterized in *T. brucei*, where they are required for normal cell growth and 18S rRNA maturation,^54^ reinforcing their status as widely conserved biogenesis factors across eukaryotes.

TbUTP6, our second bait protein, exhibited moderate overall structural conservation across examined species. The highest similarity was observed between *T. brucei* and *T. cruzi* orthologs (TM-score = 0.67), yet even this comparison displayed only moderate alignment coverage (∼53%) (Figure 4). FoldSeek^67^ and CATH-Gene3D^68^ analyses identified a tetratricopeptide-repeat (TPR) domain, consistent with its proposed role as a scaffolding protein.^104,105^ Indeed, UTP6 has been implicated in early SSU assembly as an rRNA-stabilizing chaperone and as a recruitment platform for additional factors, including components of the nuclear exosome.^21,51^

*T. brucei* UTP6 comprises two α-helical domains (domain 1: residues 1–492; domain 2: residues 544–894). *S. cerevisiae* UTP6 contains only one helical domain, comparable in size and structure to the first domain of TbUTP6, whereas in *H. sapiens*, both domains are shorter compared with the *T. brucei* ortholog (domain 1: residues 1–363; domain 2: residues 369–593) (Figure S4A). Among kinetoplastids, *Leishmania* spp. UTP6 displays a markedly expanded first domain (∼575 residues) (Figure S4B). The two domains are connected by a linker that is substantially longer in trypanosomatids (50–70 residues) than in humans (4 residues) (not shown), suggesting increased flexibility and a more variable relative orientation between domains in trypanosomatid homologs. These pronounced size differences and modular rearrangements point to species-specific remodeling of UTP6, most likely as an adaptation to the unique structural requirements of trypanosomatid pre-rRNA, including their unusually large SSU expansion segments ES6S and ES7S.^37,38^

In addition, several RBFs showed low structural conservation (0.2 ≤ TM-score <0.5) or no meaningful similarity (TM-score <0.2) when compared across species. This pattern was particularly evident for large architectural proteins such as UTP14 and NOP14, which span broad regions of the early SSU processome. UTP14 is composed of α-helices connected by extensive disordered regions (Figure S5). NOP14, however, displays a mixed pattern of conservation: although its extended α-helical regions show limited structural overlap between species, a central leucine-rich repeat-like domain of ∼250 amino acids aligns with high confidence (domain-restricted TM-scores >0.65 across all species; Figure S6). This conserved domain mediates binding to NOC4 in preribosomal particles.^20,22,23^ Notably, the *T. brucei* NOC4 homolog was enriched in both the TbUTP6 and TbPNO1 pull-downs.

Such intrinsic flexibility, characteristic of a number of large RBFs, is essential for mediating long-range contacts within preribosomal assemblies, helping to maintain structural cohesion while regulating premature association or dissociation of other factors.^22,26,27^ These observations are particularly relevant given the unusually large expansion segments in trypanosomatid 18S and 28S rRNAs, which likely require additional flexibility and expanded interaction surfaces for proper stabilization and folding.

Other highly flexible factor is LTV1 (Figure S7), whose structural alignment yielded very low TM-scores across the species analyzed (Figure 4). LTV1 is an RBF required for stabilization and proper binding of ribosomal proteins in the head and beak regions of the ribosome during biogenesis, and also plays a role in nuclear export to the cytoplasm.^26,27,106^ At the sequence level, *T. brucei* LTV1 shows similarity mainly to its predicted ortholog in *H. sapiens* and other trypanosomatids, while containing a higher proportion of positively charged residues compared with its *S. cerevisiae* ortholog (not shown). Interestingly, LTV1 binds in the vicinity of ES9S and ES10S, expansion segments that are longer in *T. brucei* than in *S. cerevisiae* and *H. sapiens*.^26,27,37,106^ A distinctive feature of *T. brucei* LTV1 is an N-terminal extension of ∼100 residues, which likely contributes to its structural diversity. Furthermore, additional residues, particularly positively charged ones, may assist in stabilizing the longer RNA expansion segments in *T. brucei*, potentially acting as molecular chaperones.

Finally, some very large RBFs, such as UTP10, and UTP20, could not be reliably aligned due to pronounced size differences among species (Figure 4). UTP10 and UTP20 are substantially expanded in trypanosomatids (*T. brucei*: 2631 and 3901 residues; *T. cruzi*: 2609 and 3915 residues; *Leishmania* spp.: ∼2770 and ∼4180 residues, respectively) compared with their counterparts in *S. cerevisiae* (1769 and 2493 residues) and *H. sapiens* (2144 and 2785 residues). These long trypanosome-specific extensions may create additional surfaces for protein–protein or protein–RNA interactions. Notably, both UTP10 and UTP20 were enriched only in the TbUTP6 pull-down, consistent with their proposed roles in early SSU assembly (Table 1). By contrast, RRP5 is strikingly shorter in trypanosomatids (*T. brucei*: 679 residues; *T. cruzi*: 669 residues; *Leishmania* spp.: ∼740 residues) than in yeast or humans (*S. cerevisiae*: 1729 residues; *H. sapiens*: 1871 residues), suggesting that distinct structural solutions may have evolved to support equivalent functions in pre-rRNA stabilization and compaction.

### Identification of Candidate Trypanosomatid-Specific Ribosome Biogenesis Factors

A subset of 29 proteins enriched in the TbUTP6 or TbPNO1 pull-downs lacked identifiable orthologs in yeast or humans and had no assigned molecular function (Figure 5). Among these, Tb927.7.650 (NOP105) and Tb927.6.630 were the only two proteins enriched with both TbUTP6 and TbPNO1, suggesting a stable association with preribosomal intermediates spanning early and intermediate stages of SSU maturation. Structural analyses revealed that both proteins adopt a conserved seven-bladed β-propeller architecture coupled to a variable C-terminal helical domain (Figure S8A,B). A similar domain organization was observed for Tb927.10.1560 (NOP91), which was enriched exclusively in the TbUTP6 pull-down (Figure S8C). FoldSeek^67^ searches identified structural similarity between TbNOP105 and UTP5,^25,45^ whose ortholog has remained elusive in trypanosomatids at the sequence level, raising the possibility that TbNOP105 represents a diverged UTP5 counterpart. Consistent with this interpretation, TrypTag data^107^ localize TbNOP105 to the nucleolus, in agreement with the expected subcellular localization of a core SSU processome component. Notably, NOP105 was also detected in proximity to spindle-associated structures in a proximity-labeling study,^90^ suggesting potential spatial or regulatory connections between preribosomal assembly and the mitotic machinery.

**Figure 5:**
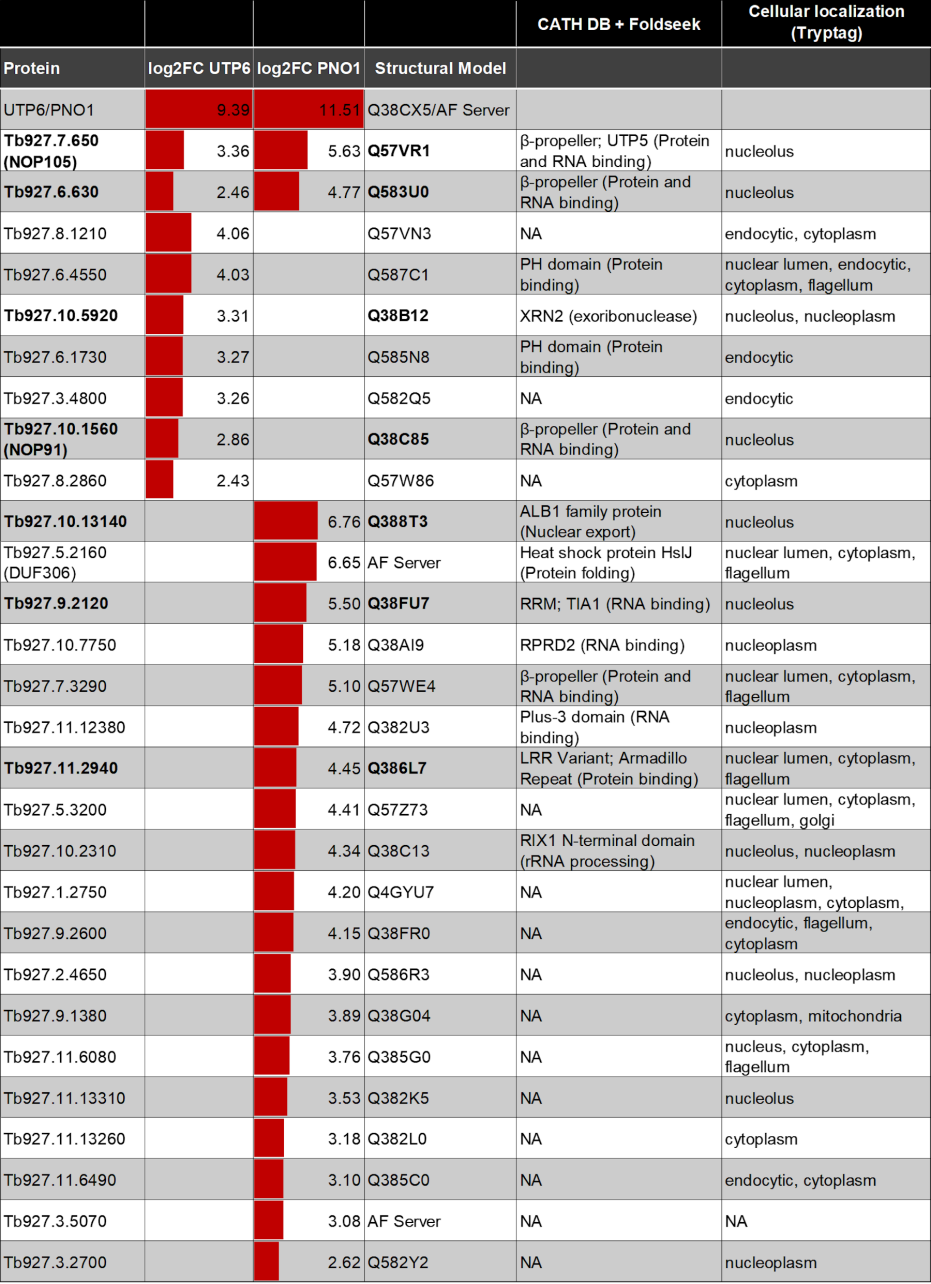
Uncharacterized proteins enriched with TbUTP6 and TbPNO1 and their predicted features. List of the 29 proteins lacking annotated function or recognizable yeast or human orthologs that were significantly enriched in TbUTP6 or TbPNO1 pull-downs. Log_2_ fold-change (log_2_ FC) values were calculated as described in the Experimental Section, and bar plots represent normalized log_2_ FC values, with the bait protein value set as maximum and 0 as minimum. Indicated structural features are based on domain analysis performed with CATH-Gene3D^68^ using an *E*-value threshold ≤0.005, and structural homology searches conducted with FoldSeek^67^ using an *E*-value threshold ≤5 × 10^–5^; in both cases, the top two hits from PDB or AlphaFoldDB were considered. “NA” indicates that no entry met the search criteria. Subcellular localization was assigned based on TrypTag data.^107^ The proteins proposed as the strongest candidates for trypanosomatid-specific RBFs (discussion in the text) are highlighted in bold.

Structural comparison across kinetoplastids of the uncharacterized proteins detected in the pull-downs revealed a wide range of TM-scores, indicating varying degrees of structural conservation and divergence among these putative trypanosomatid-specific factors (Figure 6). Several proteins exhibited consistently high TM-scores and well-defined conserved domain architectures, notably Tb927.10.5920 and Tb927.11.2940.

**Figure 6:**
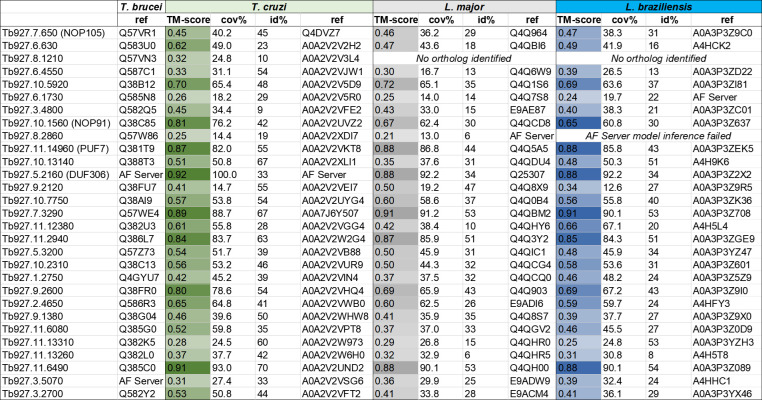
Analysis of structural conservation of uncharacterized proteins enriched with TbUTP6 or TbPNO1 across kinetoplastids. Predicted structures from the AlphaFold Database or generated using the AlphaFold server (as indicated by the codes in the column “ref”) were compared to assess structural conservation of uncharacterized proteins enriched in the UTP6 or PNO1 pull-downs. Each *T. brucei* protein was aligned using TM-align against its ortholog in *T. cruzi* (Dm28c), *L. major* (Friedlin), and *L. braziliensis* (MHOM/BR/75/M2904). TM-score, alignment coverage (cov %), and sequence identity (id %) were computed using TM-align. Color scales indicate relative TM-score values (light colors = low conservation; dark colors = high conservation).

Tb927.10.5920 is predicted to localize to the nucleolus according to TrypTag data^107^ (Figure 5). Structural prediction revealed the presence of a conserved 5′–3′ exoribonuclease (XRN) domain together with extensive disordered regions (AlphaFold Database entry Q38B12). Structural alignment of the predicted *T. brucei* Tb927.10.5920 model with the crystal structures of XRN1 from *Drosophila melanogaster* (PDB 2Y35) and Rat1 (XRN2) from *S. cerevisiae* (PDB 9Q6V) yielded TM-scores of approximately 0.45. In particular, six of the seven acidic residues involved in magnesium coordination and catalytic activity in canonical XRN enzymes^108^ are conserved in Tb927.10.5920 (not shown), suggesting partial conservation of the catalytic core.

In trypanosomatids, four XRN-family ribonucleases (XNRA-D) were initially described. However, none of them appear to be a functional Rat1 homolog with respect to the role of this enzyme in rRNA processing.^109^ Subsequently, two additional and highly divergent members, XNRE and XRNF, were identified. Whereas XRNF has not yet been assigned a specific cellular function, XRNE was shown to be required for 18S rRNA maturation and 5′-end processing of 5.8S rRNA.^110^ Considering these observations, we speculate that Tb927.10.5920 may represent a previously unrecognized XRN-like factor with a potential role in rRNA processing in *T. brucei*.

Finally, the predicted structure of Tb927.11.2940 (AlphaFold Database entry Q386L7) shows an armadillo-repeat domain, a scaffold associated with diverse protein–protein interaction networks.^111^ Several other enriched uncharacterized proteins contain RNA-recognition motifs (RRM) or Plus-3 domains, both commonly linked to RNA binding and ribosome assembly pathways (Figure 5), supporting their candidacy as novel SSU biogenesis factors. By contrast, proteins with low structural conservation across trypanosomatids, weak enrichment, and non-nuclear localization, such as Tb927.8.2860, Tb927.3.5070, and Tb927.11.13260, are unlikely to represent *bona fide* SSU assembly factors and instead likely correspond to nonspecific contaminants.

Integrating quantitative enrichment, domain composition, structural conservation, and subcellular localization from the TbUTP6 and TbPNO1 pull-downs allows us to propose seven proteins as the strongest candidates for trypanosomatid-specific RBFs: Tb927.7.650 (NOP105), Tb927.10.5920, Tb927.10.1560 (NOP91), Tb927.6.630, Tb927.10.13140, Tb927.9.2120, and Tb927.11.2940. Together, these proteins may constitute a previously unrecognized cohort of potential SSU assembly factors that may contribute to the specific features of ribosome biogenesis in trypanosomatids.

## Conclusions

In this study, we used two factors involved in small-subunit ribosome assembly as affinity baits to identify proteins participating in ribosome biogenesis in *T. brucei*. UTP6 marks early nucleolar stages, while PNO1 is associated with early- to-late maturation steps, generating two complementary sets of data. The two data sets revealed a conserved core of known ribosome biogenesis factors, together with notable divergences likely linked to the distinctive architecture of trypanosomatid rRNA. A substantial group of enriched proteins lacked recognizable orthologs outside kinetoplastids. By integrating enrichment patterns, predicted structural features, conservation within kinetoplastids, and subcellular localization, we propose seven of these uncharacterized proteins as strong candidates for trypanosomatid-specific components of small-subunit maturation. Additionally, the copurification of kinetochore- and cytokinesis-associated proteins with PNO1 suggests potential spatial or regulatory connections between ribosome biogenesis and mitotic processes, although their functional relevance remains to be determined.

Together, these findings support a model in which *T. brucei* preserves core principles of small-subunit assembly while relying on a complementary set of species-specific factors. Functional studies will now be essential to define the roles of these candidates and to determine whether they contribute to trypanosome-specific mechanisms of ribosome biogenesis.

## Supplementary Material







## Data Availability

The mass spectrometry proteomics data have been deposited in the PRIDE Archive (http://www.ebi.ac.uk/pride/archive/) via the PRIDE partner repository. The data set identifiers for the TbUTP6 and TbPNO1 pull-downs are PXD074182 and PXD074183, respectively.

## Notes

Table S1: Synthetic oligonucleotide sequences used for *T. brucei* homologous recombination. Table S2: Synthetic oligonucleotide sequences used in confirmatory polymerase chain reactions. Figure S1. Confirmation of tagging of endogenous *T. brucei* UTP6 and PNO1 proteins. Figure S2. Volcano plots of all proteins identified by LC–MS/MS. Figure S3. Structural conservation of the ribosome biogenesis factor PNO1 across kinetoplastids, yeast, and humans. Figure S4. Structural comparison of the ribosome biogenesis factor UTP6 across kinetoplastids, yeast, and humans. Figure S5. Structural comparison of the ribosome biogenesis factor UTP14 across kinetoplastids, yeast, and humans. Figure S6. Structural comparison of the ribosome biogenesis factor NOP14 across kinetoplastids, yeast, and humans. Figure S7. Structural comparison of the ribosome biogenesis factor LTV1 across kinetoplastids, yeast, and humans. Figure S8. Structural conservation of the β-propeller-containing uncharacterized proteins Tb927.7.650, Tb927.6.630 and Tb927.10.1560 across kinetoplastids (PDF)
Complete TbUTP6 pull-down data (XLSX)
Complete TbPNO1 pull-down data (XLSX)
