# Supplementary material for: Proteomic Identification of Small-Subunit Ribosome Assembly Factors in Trypanosoma brucei
Source: J Proteome Res. 2026 May 18;25(6):3118–35. doi: 10.1021/acs.jproteome.6c00066 (PMC13248013; doi:10.1021/acs.jproteome.6c00066)
Supplement: Supplementary file 1 [file pr6c00066_si_001.pdf]

Supplementary Material

Proteomic Identification of Small-Subunit Ribosome Assembly Factors in *Trypanosoma brucei*

Gustavo Guadagnini Perez [1,2], Priscila Mazzocchi Hiraiwa [1], Verônica Santana da Silva [1], Nilson Ivo Tonin Zanchin [1], Beatriz Gomes Guimarães [1,2*]

[1] Carlos Chagas Institute, Oswaldo Cruz Foundation, Rua Prof. Algacyr Munhoz Mader, 3775, 81350-010, Curitiba, PR, Brazil

[2] Biochemistry Postgraduate Program, Federal University of Paraná, Curitiba, Brazil.

* Correspondence: beatriz.guimaraes@fiocruz.br

Table of contents

Table S1: Synthetic oligonucleotide sequences used for *T. brucei* homologous recombination.

Table S2: Synthetic oligonucleotide sequences used in confirmatory polymerase chain reactions.

Supporting Table S3: Complete TbUTP6 pull-down data (provided as a separate Excel file).

Supporting Table S4: Complete TbPNO1 pull-down data (provided as a separate Excel file).

Figure S1. Confirmation of tagging of endogenous *T. brucei* UTP6 and PNO1 proteins.

Figure S2. Volcano plots of all proteins identified by LC–MS/MS.

Figure S3. Structural conservation of the ribosome biogenesis factor PNO1 across kinetoplastids, yeast, and humans.

Figure S4. Structural comparison of the ribosome biogenesis factor UTP6 across kinetoplastids, yeast, and humans.

Figure S5. Structural comparison of the ribosome biogenesis factor UTP14 across kinetoplastids, yeast, and humans.

Figure S6. Structural comparison of the ribosome biogenesis factor NOP14 across kinetoplastids, yeast, and humans.

Figure S7. Structural comparison of the ribosome biogenesis factor LTV1 across kinetoplastids, yeast, and humans.

Figure S8. Structural conservation of the β-propeller–containing uncharacterized proteins Tb927.7.650, Tb927.6.630 and Tb927.10.1560 across kinetoplastids.

Supplementary Table S1: Sequences of the synthetic oligonucleotides used for homologous recombination in *T. brucei*.

**Name**	**Sequence 5' – 3'**
TbUTP6C_F	GGCGAGCGAAGGAGGTGTCGGGACGGGCGCGAAGGGAGA CACTGGCGCCACAACATCTACTTCTCATGTTGAATAAATACGC TTCCTCTTACACGGCTCC
TbUTP6C_R	AAAGACCCGCTTTATTTAATTTTTTTAAAAAAAGAGATGATGTGG CGCTGAATGGTTACACTAGCGTCATTTACTCGCGACCAATTTG AGAGACCTGTGCG
TbPNO1N_F	TCGCTCTGTTATCGTTTACCTACCCATTTATTTGCATTTTATCGA TACTCGGCATTGAAGTGACGTGCATCTTAGGTTAACCCTAAAG TCGAGGAGGTTGAC
TbPNO1N_R	GGTTCGTCATTACCGTTGCTGCCTGTCATGGTGTTGTTAGATTT TCTCTTACCCGCCACGGCAGCAGCTGCTGAGAGCATGCTAC CGCCCAGACCGG

Supplementary Table S2: Sequences of the synthetic oligonucleotides used in polymerase chain reactions.

**Name**	**Sequence 5' – 3'**
TbUTP6_F	GGCAGTACGGTTAGCAGTCG
p2Tag_D_Rev	CCGAAGGATAGGCACCGC
TbPNO1_R	CTACACTGCGTCCCCGTAAG
p2Tag_A_Fw	CCTGTACTTCCAGTCCGAGC

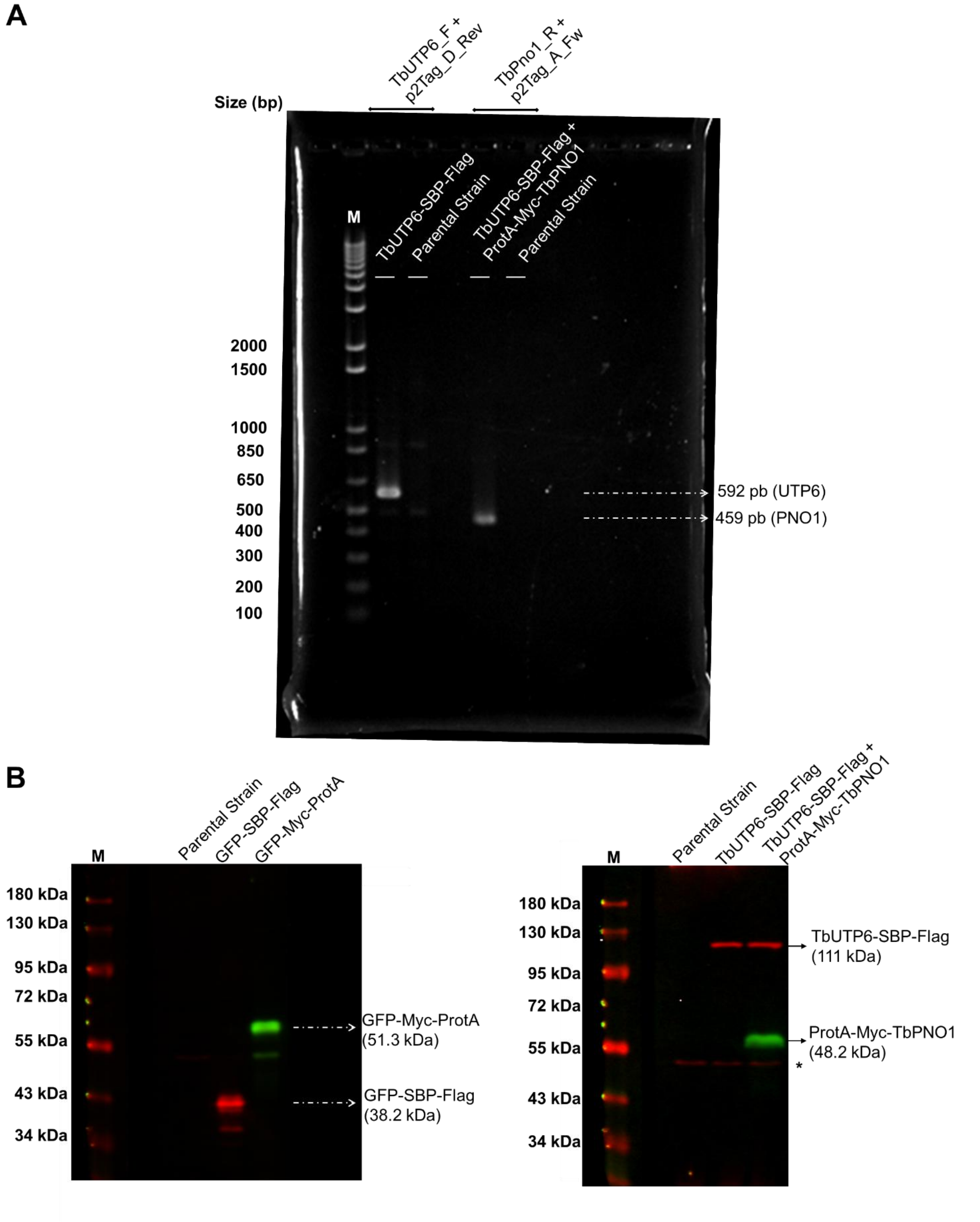

Supplementary Figure S1. Confirmation of tagging of endogenous *T. brucei* UTP6 and PNO1 proteins. (A) Agarose gel showing the PCR products obtained by using the gene- and tag-specific primers listed in Supplementary Table S2 and DNA from the strains TbUTP6–SBP–FLAG and TbUTP6–SBP–FLAG + ProtA–Myc–TbPNO1, as indicated at the top of each lane. Parental strain

indicates the DNA from the *Trypanosoma brucei brucei* Lister 427 parental strain. Presence of the PCR product indicates correct integration of the SBP-FLAG and ProtA-Myc tags at the endogenous TbUTP6 and TbPNO1 loci. Lane M corresponds to the 1 Kb Plus DNA Ladder (Invitrogen). (B) Western blot analysis confirming expression of the tagged proteins using anti-FLAG and anti-Protein A antibodies, as described in the Experimental Procedures. Identification of the strains expressing the respective tagged proteins is indicated at the top of each lane; Parental strain indicates cell extracts from the *Trypanosoma brucei brucei* Lister 427 parental strain. Lane M corresponds to the PageRuler™ Prestained Protein Ladder (Thermo Scientific). The asterisk indicates an unspecific band present in all samples using the anti-FLAG antibody.

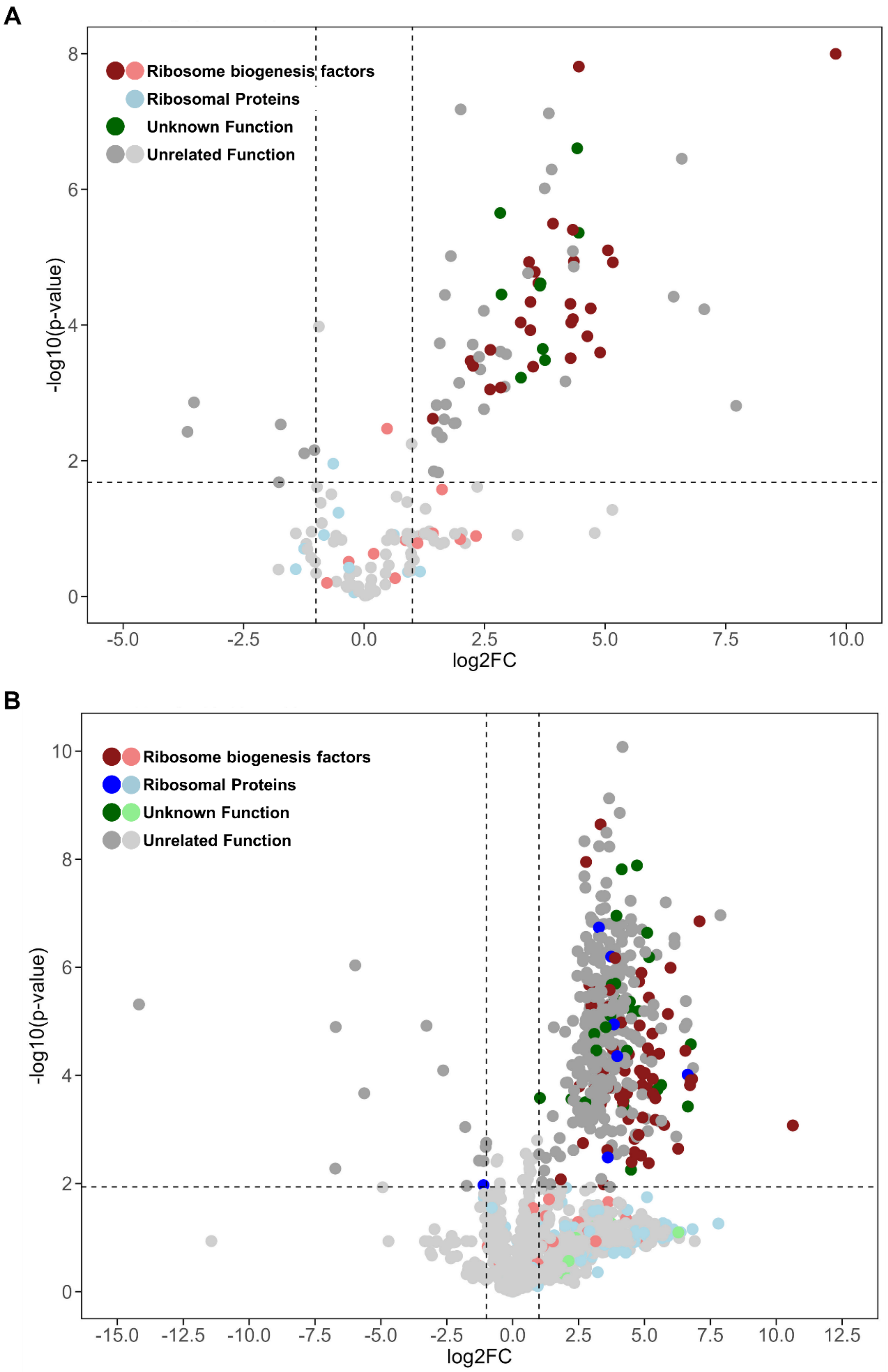

Supplementary Figure S2. Volcano plots of all proteins identified by LC–MS/MS. Volcano plots display all proteins detected in TbUTP6–SBP–FLAG (A) and ProtA–Myc–TbPNO1 (B) pull-downs, plotted as $\log_2$ fold change relative to the corresponding GFP-tagged controls (*x*-axis) versus $-\log_{10}$ *p*-value (*y*-axis). Statistical thresholds correspond to adjusted p ≤ 0.05 (FDR ≤ 0.05)

and $log_2FC > 1$. Conserved ribosome biogenesis factors are shown in red (significant) or pink (not significant); ribosomal proteins in blue (significant) or cyan (not significant); and proteins of unknown function in green (significant) or light green (not significant). Proteins of unrelated function are indicated in grey (significant) and light grey (not significant).

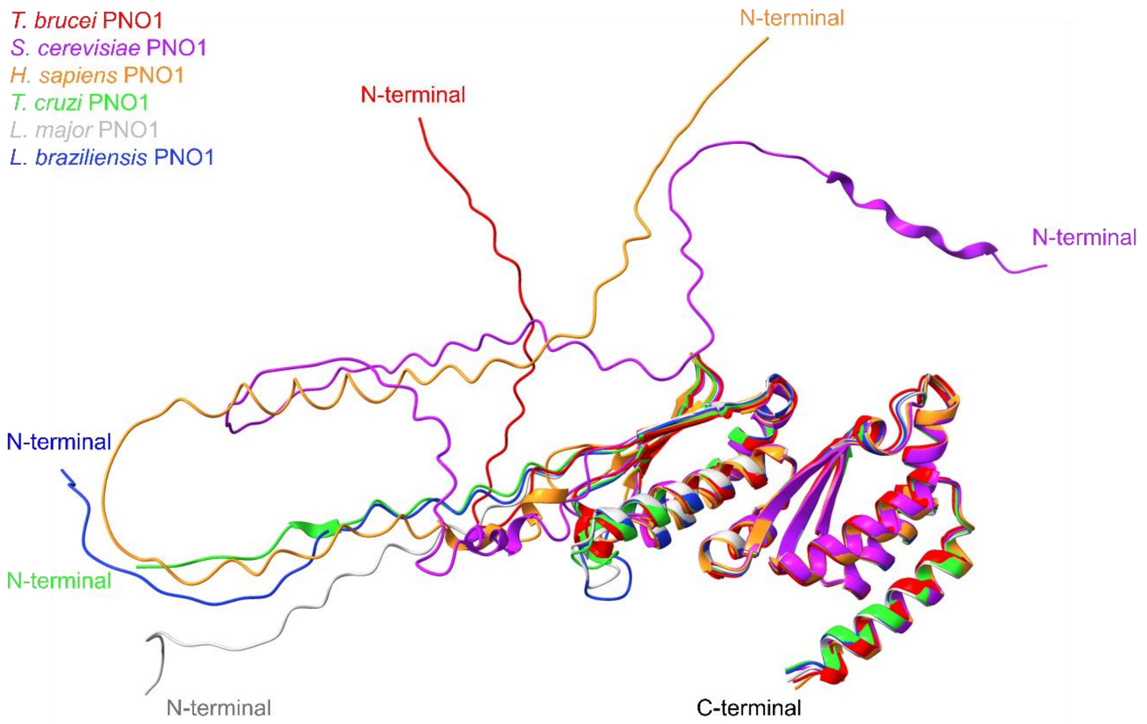

Supplementary Figure S3. Structural conservation of the ribosome biogenesis factor PNO1 across kinetoplastids, yeast, and humans. AlphaFold-predicted structures of PNO1 from *T. brucei* (red, 217 residues, generated using the AlphaFold Server), *S. cerevisiae* (pink, 274 residues, AlphaFold ID Q99216), *H. sapiens* (orange, 252 residues, AlphaFold ID Q9NRX1), *T. cruzi* (green, 211 residues, AlphaFold ID A0A2V2XN29), *L. major* (grey, 221 residues, AlphaFold ID E9AFH8), and *L. braziliensis* (blue, 221 residues, AlphaFold ID A4HN23) were aligned using TM-align and visualized in ChimeraX.

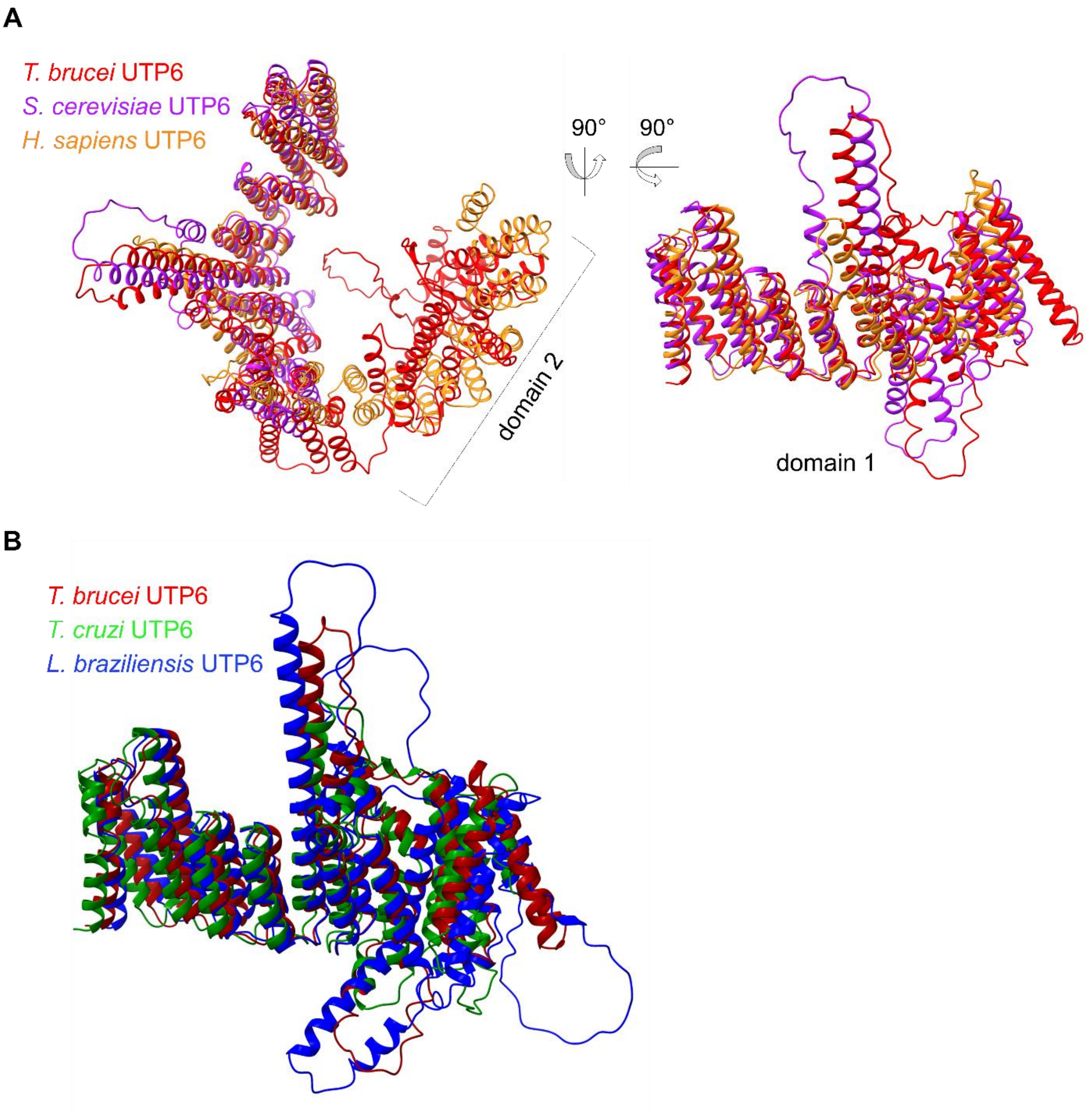

Supplementary Figure S4. Structural comparison of the ribosome biogenesis factor UTP6 across kinetoplastids, yeast, and humans. (A) Superposition of AlphaFold-predicted structures of *T. brucei* UTP6 (red, 897 residues, AlphaFold ID Q38CX5), *S. cerevisiae* UTP6 (purple, 440 residues, AlphaFold ID Q02354), and *H. sapiens* UTP6 (orange, 597 residues, AlphaFold ID Q9NYH9). The left panel highlights the absence of the second helical domain in the yeast homolog (purple). The right panel shows the superposition of the first helical domain, highlighting the shorter helices present in the human homolog (orange). (B) Superposition of the first helical domains of *T. brucei* UTP6 (red, 1-492 residues, AlphaFold ID Q38CX5) *T. cruzi* UTP6 (green, 1-455 residues, AlphaFold ID A0A2V2UYM9), and *L. braziliensis* UTP6 (blue, 1-568 residues, AlphaFold ID A0A3P3ZH12). Structural alignments were carried out using TM-align, and visualizations were generated in ChimeraX.

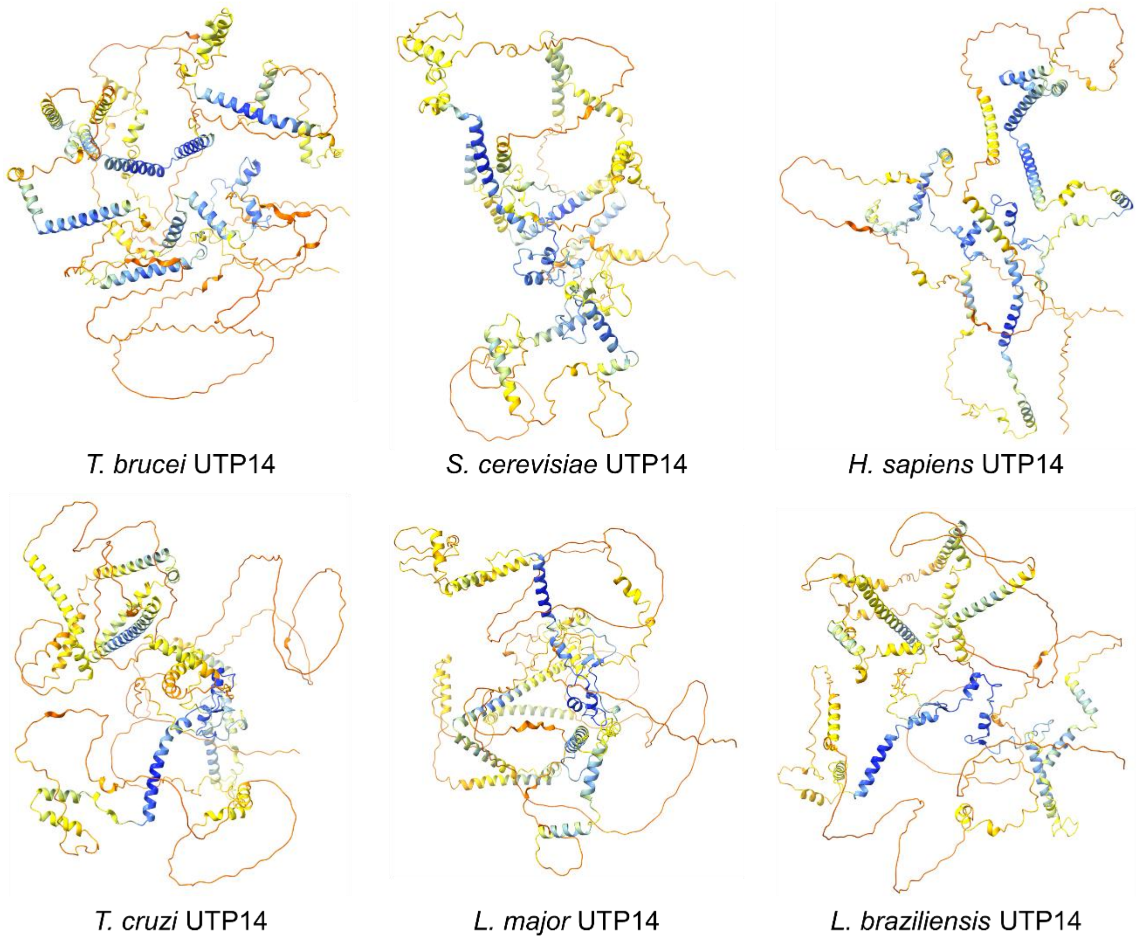

Supplementary Figure S5. Structural comparison of the ribosome biogenesis factor UTP14 across kinetoplastids, yeast, and humans. AlphaFold-predicted structures of UTP14 from *T. brucei* (1040 residues, AlphaFold ID Q388G9), *S. cerevisiae* (899 residues, AlphaFold ID Q04500), *H. sapiens* (766 residues, AlphaFold ID Q5TAP6), *T. cruzi* (1052 residues, AlphaFold ID A0A7J6YG53), *L. major* (999 residues, AlphaFold ID Q4Q5S1), and *L. braziliensis* (993 residues, AlphaFold ID A4HJZ5) were visualized in ChimeraX. Structures are colored according to AlphaFold pLDDT (predicted local distance difference test) scores, with dark blue indicating regions of high confidence and yellow/orange indicating regions of low confidence. The predicted models reveal an extensive degree of intrinsic disorder and structural flexibility across species.

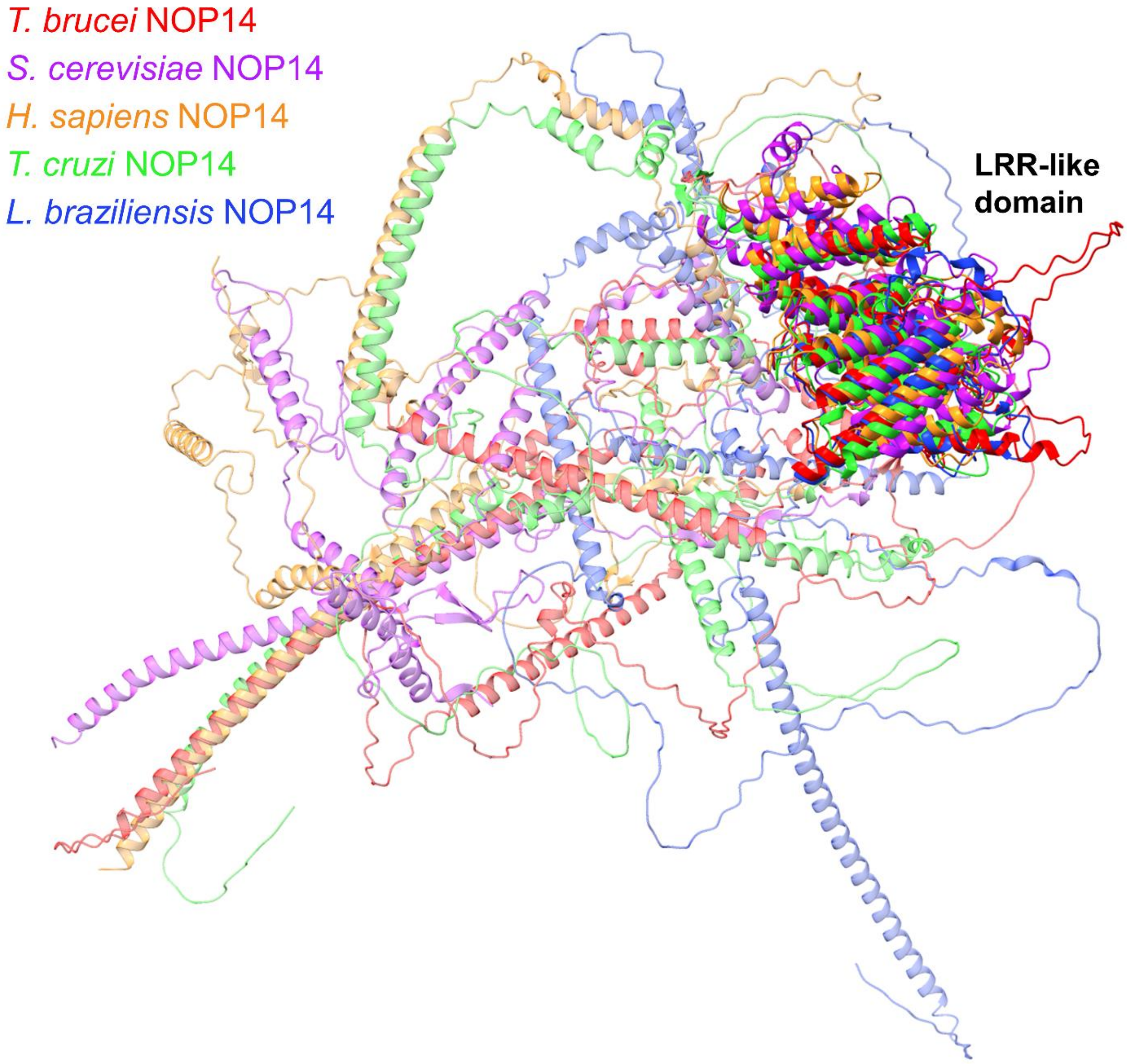

Supplementary Figure S6. Structural comparison of the ribosome biogenesis factor NOP14 across kinetoplastids, yeast, and humans. AlphaFold-predicted structures of NOP14 from *T. brucei* (red, 955 residues, generated using the AlphaFold Server), *S. cerevisiae* (pink, 899 residues, AlphaFold ID Q99207), *H. sapiens* (orange, 857 residues, AlphaFold ID P78316), *T. cruzi* (green, 892 residues, AlphaFold ID A0A2V2V9E6), and *L. braziliensis* (blue, 906 residues, AlphaFold ID A0A3P3YWY4) were aligned using TM-align based on the conserved central domain and visualized in ChimeraX. The superposition highlights the structural similarity of the leucine-rich-repeat–like domain (corresponding to residues 492–821 in *T. brucei* NOP14). TM-scores and sequence identities relative to *T. brucei* NOP14 for this aligned domain are as follows: *S. cerevisiae* (residues 363–694: TM-score 0.68; 15% identity), *H. sapiens* (residues 427–745: TM-score 0.68; 17% identity), *T. cruzi* (residues 476–767: TM-score 0.82; 66% identity), and *L. braziliensis* (residues 487–782: TM-score 0.82; 39% identity).

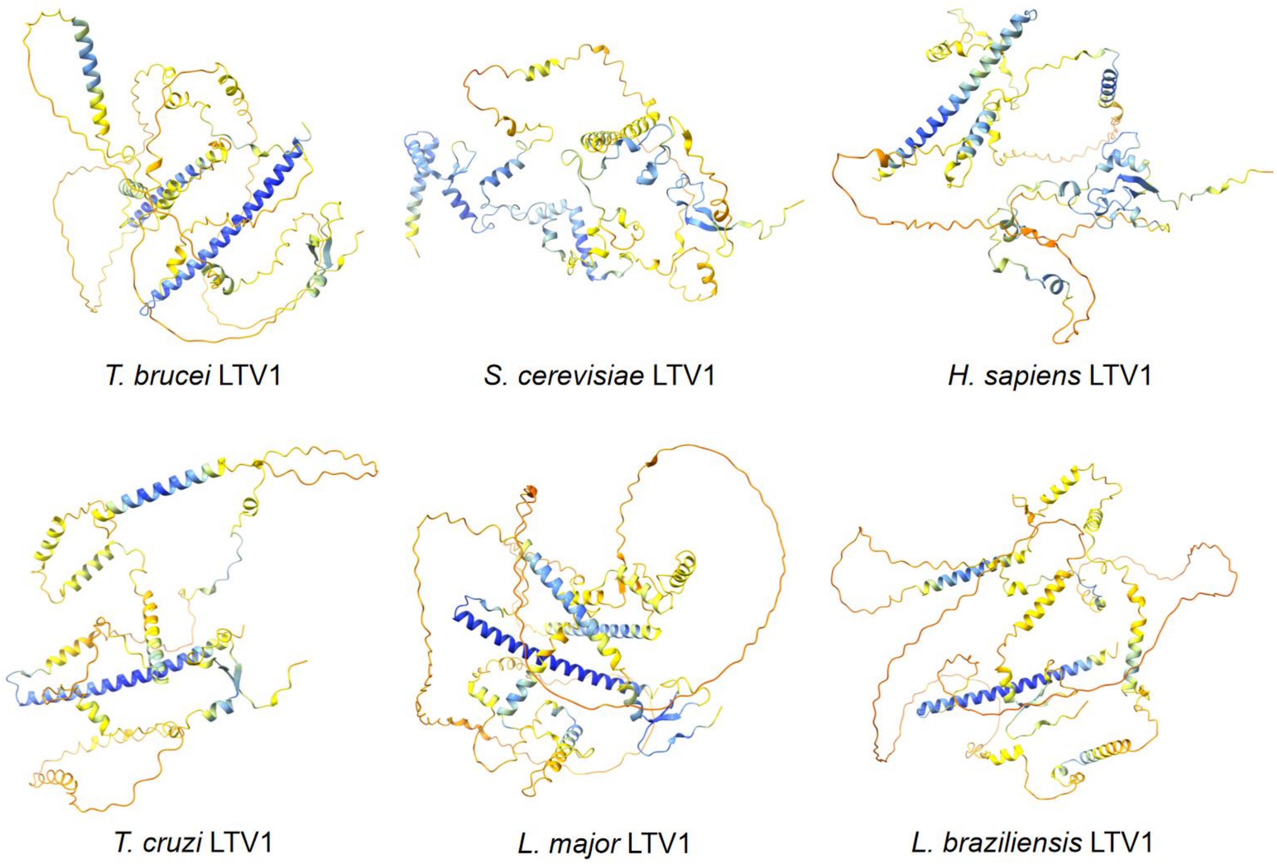

Supplementary Figure S7. Structural comparison of the ribosome biogenesis factor LTV1 across kinetoplastids, yeast, and humans. AlphaFold-predicted structures of LTV1 from *T. brucei* (550 residues, AlphaFold ID Q57WD1), *S. cerevisiae* (463 residues, AlphaFold ID P34078), *H. sapiens* (475 residues, AlphaFold ID Q96GA3), *T. cruzi* (457 residues, AlphaFold ID A0A2V2VRS8), *L. major* (658 residues, AlphaFold ID E9ADZ7), and *L. braziliensis* (621 residues, AlphaFold ID A4HHE8) were visualized in ChimeraX. Structures are colored according to AlphaFold pLDDT (predicted local distance difference test) scores, with dark blue indicating regions of high confidence and yellow/orange indicating regions of low confidence. The predicted models reveal an extensive degree of intrinsic disorder and structural flexibility across species.

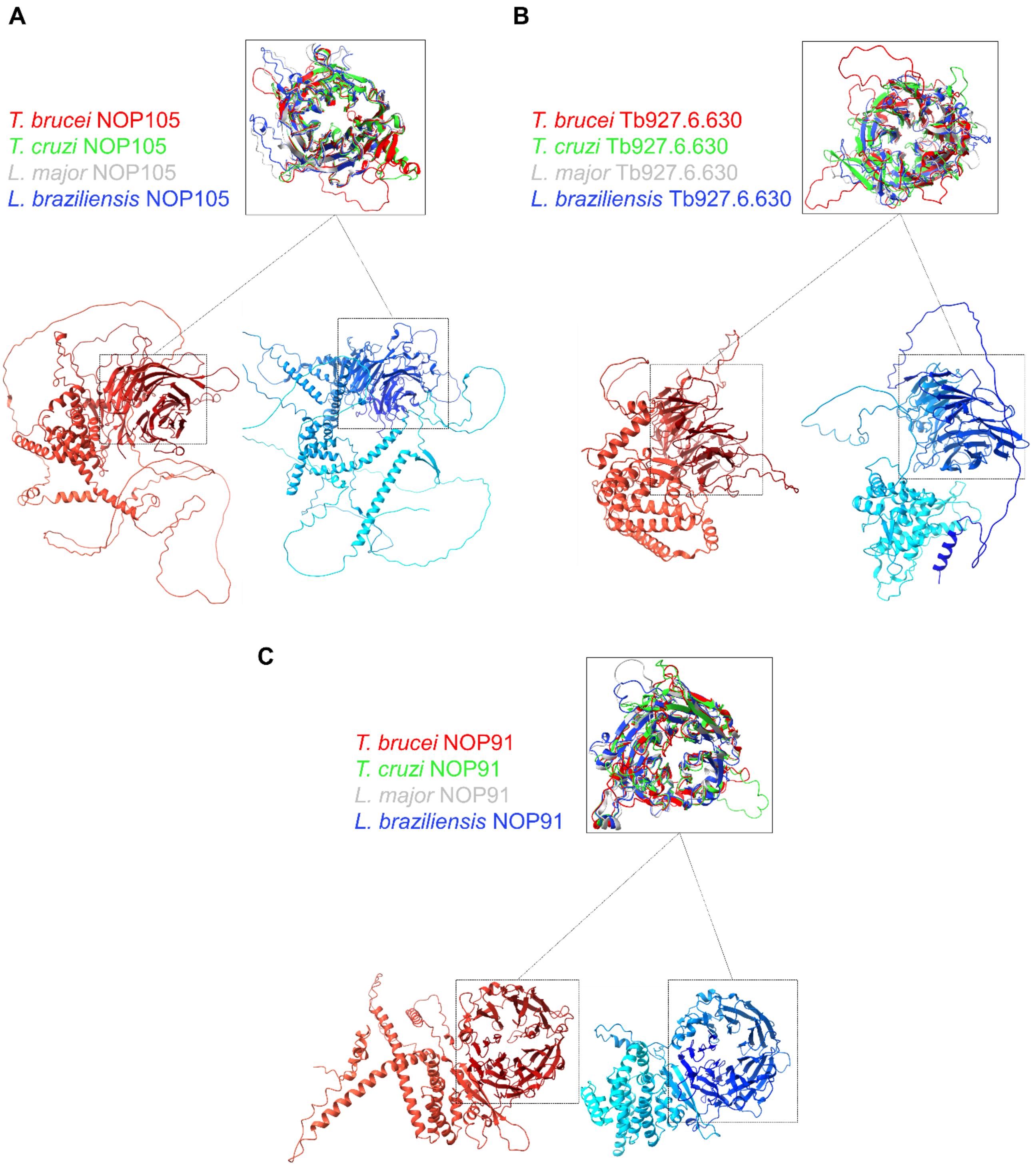

Supplementary Figure S8. Structural conservation of the β-propeller–containing uncharacterized proteins Tb927.7.650, Tb927.6.630 and Tb927.10.1560 across kinetoplastids. (A) AlphaFold-predicted structures of Tb927.7.650 (NOP105) from *T. brucei* (red, 984 residues, AlphaFold ID Q57VR1), *T. cruzi* (green, 915 residues, AlphaFold ID Q4DVZ7), *L. major* (grey, 1190 residues, AlphaFold ID Q4Q964), and *L. braziliensis* (blue, 1189 residues, AlphaFold ID A0A3P3Z9C0) were aligned using TM-align and visualized in ChimeraX. Full length structures of *T. brucei* and *L. braziliensis* homologs are shown as representatives. The inset highlights the superposition of the β-propeller domain, corresponding to residues 9-453 (*T. brucei*), 9-410 (*T. cruzi*), 17-449 (*L. major*), and 13-452 (*L. braziliensis*). (B) AlphaFold-predicted

structures of Tb927.6.630 from *T. brucei* (red, 730 residues, AlphaFold ID Q583U0), *T. cruzi* (green, 681 residues, AlphaFold ID A0A2V2V2H2), *L. major* (grey, 723 residues, AlphaFold ID Q4QBI6), and *L. braziliensis* (blue, 808 residues, AlphaFold ID A4HCK2) were aligned using TM-align and visualized in ChimeraX. Full length structures of *T. brucei* and *L. braziliensis* homologs are shown as representatives. The inset highlights the superposition of the β-propeller domain, corresponding to residues 2-402 (*T. brucei*), 29-384 (*T. cruzi*), 2-362 (*L. major*), and 104-453 (*L. braziliensis*). (C) AlphaFold-predicted structures of Tb927.10.1560 (NOP91) from *T. brucei* (red, 827 residues, AlphaFold ID Q38C85), *T. cruzi* (green, 825 residues, AlphaFold ID A0A2V2UVZ2), *L. major* (grey, 721 residues, AlphaFold ID Q4QCD8), and *L. braziliensis* (blue, 722 residues, AlphaFold ID A0A3P3Z637) were aligned using TM-align and visualized in ChimeraX. Full length structures of *T. brucei* and *L. braziliensis* homologs are shown as representatives. The inset highlights the superposition of the β-propeller domain, corresponding to residues 4-399 (*T. brucei*), 4-413 (*T. cruzi*), 5 - 381 (*L. major*), and 5-381 (*L. braziliensis*).
